# Amphiphilic Graft Copolymer-Templated Pd Nanoparticles: A Highly Recyclable Catalyst for Greener Mizoroki–Heck Reaction

**DOI:** 10.3390/ma19214434

**Published:** 2026-10-22

**Authors:** Muhammad Faizan Ali, Bungo Ochiai

**Affiliations:** Department of Chemistry and Chemical Engineering, Graduate School of Science and Engineering, Yamagata University, 4-3-16 Jonan, Yonezawa 992-8510, Yamagata, Japan; faizanmughal064@gmail.com

**Keywords:** heterogeneous Pd catalyst, amphiphilic graft copolymer, palladium nanoparticles, Mizoroki–Heck reaction, catalyst recycle, life cycle assessment, green chemistry

## Abstract

We report easily recyclable Pd nanoparticle (PdNP) catalysts embedded within an amphiphilic graft copolymer designed with dual functionality: high dispersion stability and facile isolation via centrifugation. The graft copolymer, synthesized through the reaction of poly(styrene-*alt*-maleic anhydride) with amine-terminated polyether, effectively controlled the Pd nucleation and growth of PdNPs via mild reduction with methanol. This process yielded uniform, catalytically active PdNPs integrated within the copolymer matrix. The amphiphilic polyether chains ensured dispersion stability, whereas the carboxylate moieties served as Pd-coordination sites. This polymer facilitated both the hybridization of Pd with the polymer chains and the interparticle interactions required for centrifugal precipitation. Compared with conventional heterogeneous Pd catalysts, this catalyst enabled the Mizoroki–Heck reaction at lower temperatures using butanol as a safer solvent, resulting in a 34.2% reduction in Global Warming Potential (GWP) and a 47.3% reduction in the total primary energy demand compared to the conventional DMF system. The catalyst achieved a turn over number (TON) of 64.3 and a turnover frequency (TOF) of 3.22 h^−1^. The excellent recoverability of rare Pd without loss of activity, combined with the results of a life cycle assessment, demonstrates a practical approach to recyclable heterogeneous catalysis with a reduced environmental footprint.

## 1. Introduction

The Mizoroki–Heck reaction is one of the most widely applied transformations in organic chemistry [1,2,3]. It is typically catalyzed by Pd(II)/Pd(0) species and enables the formation of C–C bonds between sp^2^-hybridized carbon atoms. In this Pd-catalyzed process, aryl halides are coupled with alkenes to produce aryl vinylene derivatives [4,5,6]. The resulting compounds are highly valuable for a broad range of applications, including medicinal chemistry, electronics, and sensing [7,8,9].

The utility of Pd catalysts in various coupling reactions is still expanding [10]. However, Pd is a rare metal with maldistribution in the Earth’s crust, and its price is rapidly increasing. This recent situation has made the development of active, recyclable catalysts highly desirable. In heterogeneous systems, Pd species are immobilized on solid supports [11,12,13,14,15]. This immobilization enables facile recovery via simple processes, such as filtration and centrifugation. In contrast, heterogeneous catalysts often suffer from inferior stability and activity issues. In heterogeneous systems, stabilizing ligands such as phosphines, typically used in homogeneous systems, are unavailable because Pd species are leached into solutions during the catalytic cycle [16,17,18,19,20]. These issues cannot be addressed without the appropriate design of efficient heterogeneous Pd catalyst systems, such as anchors that stabilize Pd species through moderate interactions without degrading the active sites on the support surface [21,22,23,24]. Furthermore, environmentally benign reaction conditions—using safer solvents and auxiliaries and design for energy efficiency—are also involved in the Principles of Green Chemistry, as well as the prevention of waste [25,26,27,28,29,30,31,32].

Amphiphilic graft copolymers have attracted considerable attention owing to their simple synthesis and tunable physicochemical properties [33,34]. For example, polymer solubility can be precisely controlled by adjusting the graft chain length or modifying the polarities of the main and graft chains [35,36,37,38,39]. In addition, functional groups capable of coordinating metal species, such as Pd, can be readily introduced into the copolymer structure [40]. Styrene–maleic anhydride copolymer (SMA) is an excellent alternating copolymer that can be functionalized via anhydride ring-opening reactions [41], generating carboxy groups along the main chain. For example, Jeffamine, a terminal amine-containing polyether, can be grafted onto SMA through amidation reactions [42]. This modification introduces amphiphilic polyether chains and additional functional groups, enhancing the dispersibility and metal-binding ability, respectively.

**Figure 1:**
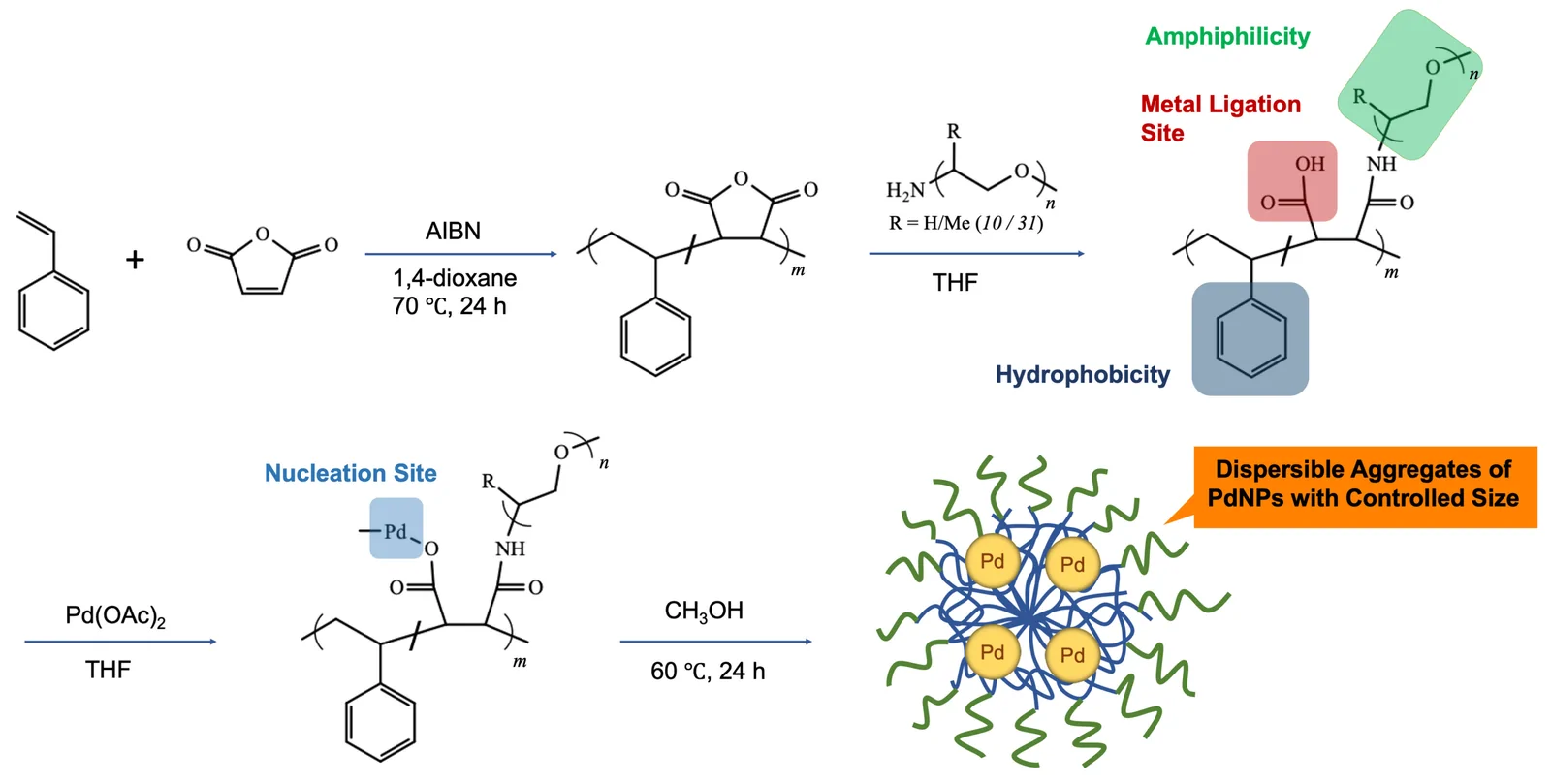
Schematic illustration of the synthesis of SMA-*g*-polyether via ring-opening amidation, followed by coordination and in situ reduction of Pd^2+^ within the polymer template.

In this study, Pd nanoparticles (PdNPs) were immobilized onto an amphiphilic SMA-*g*-polyether template matrix via a simple and efficient method, resulting in a well-dispersed and stable heterogeneous Pd nanocatalyst. The catalyst was dispersible in polar solvents and could be readily recovered by centrifugation. The prepared catalyst comprised single nano-sized PdNPs embedded within the polymer matrix (Figure 1). Its catalytic performance was evaluated in the Mizoroki–Heck reaction in 1-butanol. The catalyst exhibited excellent activity, providing high yields within short reaction times, and demonstrated good recyclability without a significant loss of performance.

It should be noted that conventional similar systems often use aprotic polar solvents, such as *N*,*N*-dimethylformamide (DMF) [43]. However, DMF has problems related to toxicity and environmental burden [44]. Furthermore, their strong polarity often reduces the efficiency of palladium recovery, causing the loss of this precious metal in every cycle. In contrast, butanol, a greener solvent with lower toxicity and greater biomass availability for production [44,45,46,47,48,49,50,51,52,53,54,55], is advantageous for achieving complete recovery of the Pd catalyst because of its lower solvation ability. To quantitatively evaluate the environmental impact of these improvements, we performed a life cycle assessment.

## 2. Materials and Methods

### 2.1. Materials

Styrene and maleic anhydride were purchased from Tokyo Chemical Industry. Styrene was purified by vacuum distillation under nitrogen prior to use. Maleic anhydride was purified by recrystallization from chloroform. 2,2′-Azobisisobutyronitrile (AIBN) was obtained from Sigma-Aldrich (St. Louis, MO, USA) and recrystallized from methanol. Jeffamine M-2070 (monoamine-terminated poly(ethylene oxide-*co*-propylene oxide) with an average unit composition of 6/35) was supplied by Huntsman Corporation (The Woodlands, TX, USA) and used as received. 1,4-Dioxane, tetrahydrofuran (THF), and methanol (superdehydrated grades) were purchased from FUJIFILM Wako Pure Chemical Corporation (Osaka, Japan). Palladium(II) acetate [Pd(OAc)_2_], iodobenzene, 4-iodoanisole, 4-iodonitrobenzene, butyl acrylate, and 1-butanol were obtained from Tokyo Chemical Industry (Tokyo, Japan). Triethylamine (Et_3_N) was purchased from Kanto Chemical Co., Inc. (Tokyo, Japan) and used without further purification. All other reagents and solvents were of analytical grade and used as received, unless otherwise noted.

### 2.2. Characterization

The unit ratios of SMA and SMA-*g*-polyether were calculated by ^1^H-NMR spectroscopic analysis (500 MHz) on a JEOL (Tokyo, Japan) JNM-EC500 spectrometer using DMSO-*d*_6_ as the solvent. Scanning electron microscopy (SEM) and bright-field scanning transmission electron microscopy (BF STEM) images were acquired using a JEOL JSM-IT800 scanning electron microscope at an acceleration voltage of 30 kV. Transmission electron microscopy (TEM) was performed using a JEOL JEM-2100F microscope operated at an accelerating voltage of 200 kV. Samples for electron microscopy were prepared by drop-casting a methanol dispersion onto carbon-coated copper grids. Gel permeation chromatography (GPC) measurements were performed on a Tosoh (Tokyo, Japan) HLC-8320GPC equipped with TSKgel SuperAW columns using DMF containing 10 mmol/L LiBr as an eluent at a flow rate of 0.6 mL/min at 40 °C based on calibration using polystyrene standards. X-ray diffraction (XRD) measurements were performed using a Rigaku (Tokyo, Japan) MiniFlex diffractometer equipped with a Cu Kα radiation source. Dynamic light scattering (DLS) measurements were performed using a Malvern (Worcestershire, UK) Zetasizer Nano-ZS instrument equipped with a 4 mW He-Ne laser (633 nm). Measurements were conducted in methanol at 25 °C using a 173° backscattering angle. Inductively coupled plasma mass spectrometry (ICP-MS) measurements were performed on a PerkinElmer ELAN DRC II spectrometer (^106^Pd, standard addition method). Samples were digested in 10 mL concentrated HNO_3_ at 150 °C and diluted to 25 mL with ultrapure water. The solutions were diluted to appropriate concentrations prior to analysis.

### 2.3. Synthesis of SMA, SMA-g-Polyether, and PdNP Catalysts

**Synthesis of SMA:** Styrene (10.0 mmol, 1.04 g) and maleic anhydride (10.0 mmol, 980 mg) were dissolved in 1,4-dioxane to obtain a 15 wt% solution in a round-bottom flask equipped with a magnetic stir bar. AIBN (1.0 mol% relative to total monomers, 33 mg) was added as the radical initiator. The reaction mixture was degassed by nitrogen bubbling and maintained under a nitrogen atmosphere. Polymerization was carried out at 70 °C for 24 h with continuous stirring. After completion, the reaction mixture was precipitated into an excess amount of cold diethyl ether. The resulting polymer was collected and redissolved in superdehydrated THF, followed by reprecipitation into diethyl ether. This reprecipitation process was repeated twice to ensure the removal of residual monomers and low-molecular-weight species. The precipitated polymer was collected by filtration and dried under vacuum at room temperature for 24 h to yield SMA as a white solid (72% yield, *M*_n_ = 213,800, *M*_w_/*M*_n_ = 386,800/213,800 = 1.81, styrene/maleic anhydride unit ratio = 51/49) (Figure S1).

**Synthesis of SMA-*g*-polyether:** For the grafting reaction, SMA (202 mg, 1.00 mmol of anhydride units) and Jeffamine M-2070 (2.00 g, 2.00 mmol; anhydride/Jeffamine molar ratio = 1:2) were dissolved in THF (5 mL) and stirred at room temperature for 24 h. The resulting SMA-*g*-polyether was purified by precipitation into a hexane/diethyl ether mixture (1/4, *v*/*v*). The product was collected and dried under vacuum for 24 h to afford 800 mg of the polymer (39.6% yield based on the initial anhydride units). Spectroscopic analysis indicated near-complete conversion of anhydride groups, consistent with an apparent Jeffamine grafting ratio of ~100% (see Figures S2–S4 for ^1^H NMR and FTIR spectra and Tables S1 and S2 for corresponding integral values).

**Preparation of PdNP Catalysts:** A predetermined amount of Pd(OAc)_2_ was placed in a three-neck flask fitted with a magnetic stir bar and a nitrogen balloon, then the flask was evacuated under vacuum using a liquid-nitrogen-cooled cold trap connected between the reaction flask and the vacuum pump, followed by nitrogen purging for three cycles to remove oxygen and establish a nitrogen atmosphere. THF (superdehydrated) was then added via syringe under a nitrogen atmosphere, and the mixture was stirred for 1 h at room temperature.

Separately, a stock solution of SMA-*g*-polyether was prepared by dissolving 300 mg of the polymer in 120 mL of methanol (superdehydrated) under nitrogen to give a polymer solution with a 2.5 mg mL^−1^ concentration. This polymer stock solution was added dropwise to the Pd(OAc)_2_ solution at room temperature with continuous stirring, maintaining a nitrogen atmosphere throughout (Table 1). The mixture was allowed to equilibrate for 3 h to promote the coordination between Pd^2+^ ions and the polymer matrix. The solution was then heated at 60 °C for 24 h, during which methanol acted as a mild reducing agent to generate PdNPs within the polymer template.

**Figure 2:**
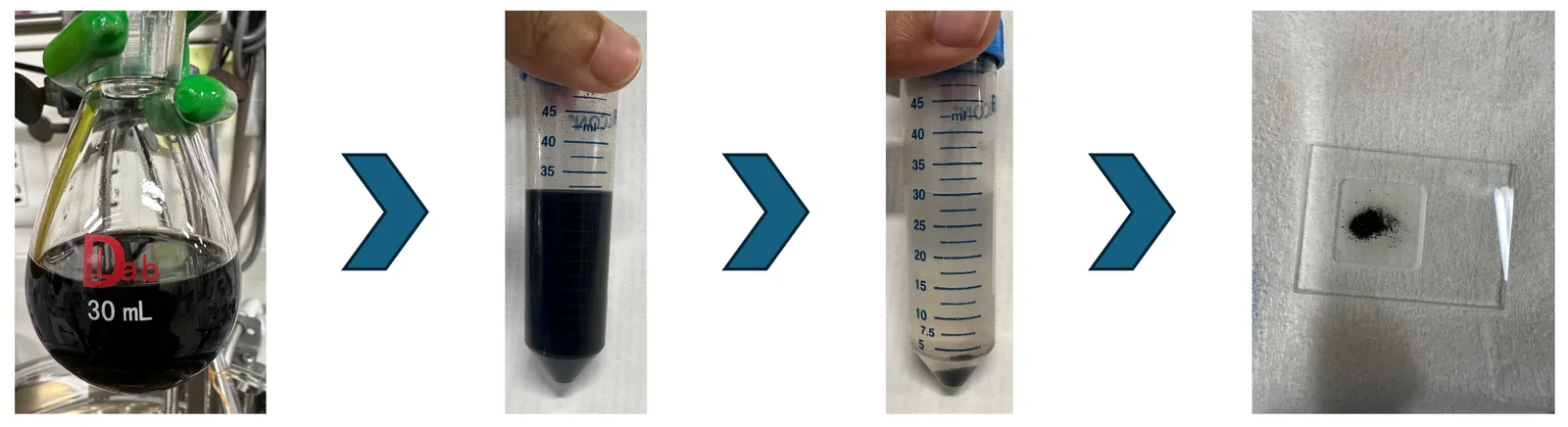
Photographs of the isolation process of embedded PdNP catalysts by centrifugation.

The resulting PdNP catalysts were isolated by centrifugation at 14,000 rpm and redispersed in methanol. This washing cycle was repeated three times to remove residual impurities. The embedded PdNP catalysts S-10, S-15, and S-20 were obtained in 92%, 90%, and 96% yields, respectively. For TEM and DLS characterizations, the purified PdNPs were redispersed in methanol by sonication (Figure 2). The preliminary structural and morphological features of the prepared catalysts (S-10, S-15, and S-20) were confirmed by TEM (Figure S5), STEM/SEM (Figure S6), XRD (Figure S7), and DLS (Figure S8).

### 2.4. Mizoroki–Heck Reaction Using PdNP Catalysts

Iodobenzene (1.00 mmol, 204 mg), *n*-butyl acrylate (2.00 mmol, 256 mg), and triethylamine (2.00 mmol, 202 mg) were dissolved in butanol (4.0 mL) in a 25 mL two-necked round-bottom flask fitted with a Dimroth condenser. The PdNP catalyst (10.2 mg for S-10, containing 77.8 μmol of Pd) was added to the solution. The flask was fitted with a nitrogen balloon, and the mixture was degassed through three cycles of evacuation via a liquid-nitrogen-cooled trap, followed by backfilling with nitrogen using a positive-displacement vacuum pump. The reaction was then carried out at 100 °C for 4 h under a nitrogen atmosphere. The conversion was determined by ^1^H NMR spectroscopy of the crude reaction mixtures (Figures S10–S13).

After the reaction, the mixture was cooled to room temperature by the addition of 10 mL of a methanol/diethyl ether (3:1, *v*/*v*) mixture. The mixture was centrifuged at 14,000 rpm for 30 min to isolate the catalyst. The supernatant was decanted, and the catalyst was washed with a methanol/diethyl ether (3:1, *v*/*v*) mixture (15 mL × 3 times) to remove residual organic compounds. The washed catalyst was dried under vacuum at room temperature for 6 h and reused in the subsequent cycle. The decanted mixture was extracted with water to remove salts, and the organic phase was concentrated under reduced pressure to obtain the product (*n*-butyl cinnamate). The isolated product was characterized by ^1^H NMR spectroscopy (Figures S14 and S15). Recycling experiments were performed under the same conditions using the recovered catalysts.

## 3. Results and Discussion

### 3.1. Synthesis and Characterization of Catalysts

The SMA-*g*-polyether template was synthesized via the ring-opening amidation of SMA with Jeffamine M-2070, followed by the coordination and in situ reduction of Pd ions within the polymer template (Figure 1).

**Figure 3:**
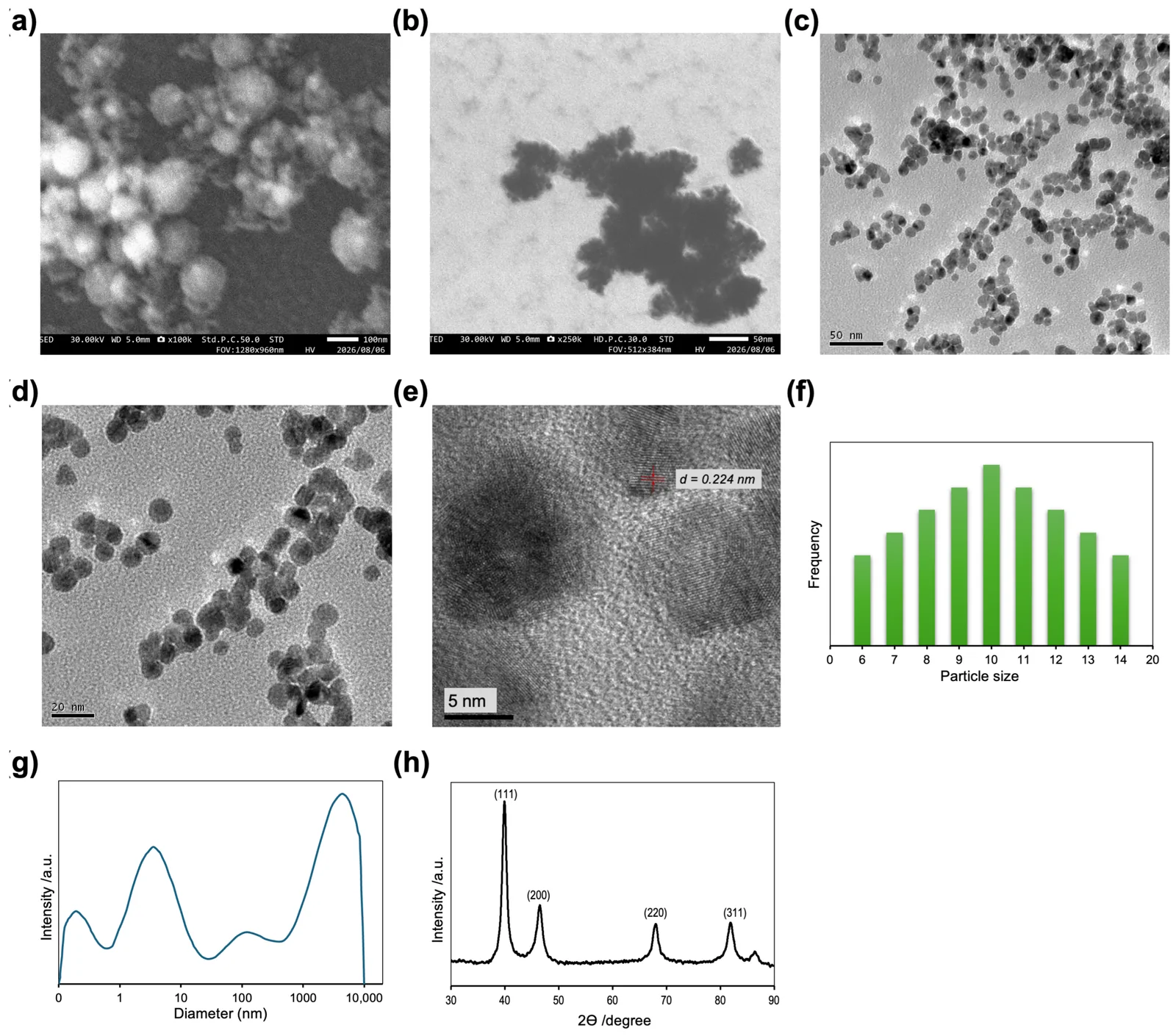
(**a**) SEM, (**b**) BF-STEM, (**c**,**d**) TEM, and (**e**) HR-TEM images; (**f**) size distribution histogram of PdNPs in the TEM image; (**g**) DLS intensity distribution; and (**h**) XRD pattern of S-10.

The SMA-*g*-polyether template enabled the formation of uniformly small Pd nanoparticles (Figure 3 and Figure S5). The SEM image of S-10 shows particles ranging 10–100 nm connected by glue-like structures, which are attributable to the polymer matrix (Figure 3a). The BF STEM image revealed the presence of high-atomic-weight nanoparticles, which are observed black and attributed to PdNPs, embedded in the light-atomic-weight matrix, which is observed gray and attributable to the polymer matrix (Figure 3b and Figure S6). TEM analysis (Figure 3c,d) clarified that the nanoparticles had an average size of approximately 10 nm. HR-TEM (Figure 3e) revealed lattice fringes with an interplanar spacing of 0.224 nm, which agrees well with the (1 1 1) lattice plane of the face-centered cubic Pd(0). The particle size distribution histogram (Figure 3f) shows a narrow size range. These images show single-nano-sized PdNPs assembled into particles in the tens of nanometers, connected by the polymer matrix.

In solution, the PdNPs did not exist as discrete nanoparticles but as microgels. The DLS profile (Figure 3g and Figure S8) shows broad peaks ranging from single nanometers to several micrometers. The smaller fraction with sizes below 10 nm corresponds to individual polymer-coated PdNPs, agreeing with the sizes of PdNPs observed in the TEM image. In contrast, the larger fraction is attributable to swollen aggregates of PdNPs connected by the polymer template.

The XRD profile (Figure 3h and Figure S7), exhibiting peaks at 40.1° (1 1 1), 46.7° (2 0 0), 68.1° (2 2 0), and 81.2° (3 1 1), agrees with the pattern of face-centered-cubic Pd(0) crystals (The parameters for XRD acquisition are expressed in Table S3, and the values for calculation of crystallite-size by Scherrer’s equation are presented in Table S4). The Pd compositions of S-10, S-15, and S-20 quantified by ICP-MS were 82.8 wt%, 94.0 wt%, and 99.0 wt%, respectively, correlating with the order of the Pd ratio in feed.

The amphiphilic nature of the polymer plays a critical role in controlling nucleation and growth. The hydrophobic styrene segments provide a confined environment for Pd nucleation, whereas the polyether chains stabilize the nanoparticles and prevent uncontrolled aggregation during formation.

These microgels are highly swellable owing to the amphiphilic polyether chains, leading to expanded structures in solutions. This swellability manifests the dispersibility of the physically crosslinked PdNP network, which is effective for catalytic reactions. The swollen microgels were efficiently precipitated by centrifugation at 14,000 rpm (Figure 2). The precipitated catalyst can be redispersed after separation. This behavior provides a simple and effective method for nanoparticle recovery without the need for filtration or complex separation techniques.

The small size of PdNPs ensures high surface accessibility, which is essential for their high catalytic activity. Simultaneously, the formation of larger microgel assemblies enables practical handling and recovery. This combination of nanoscale activity and microscale separability represents an advantage over conventional systems.

### 3.2. Mizoroki–Heck Reaction

**Figure 4:**
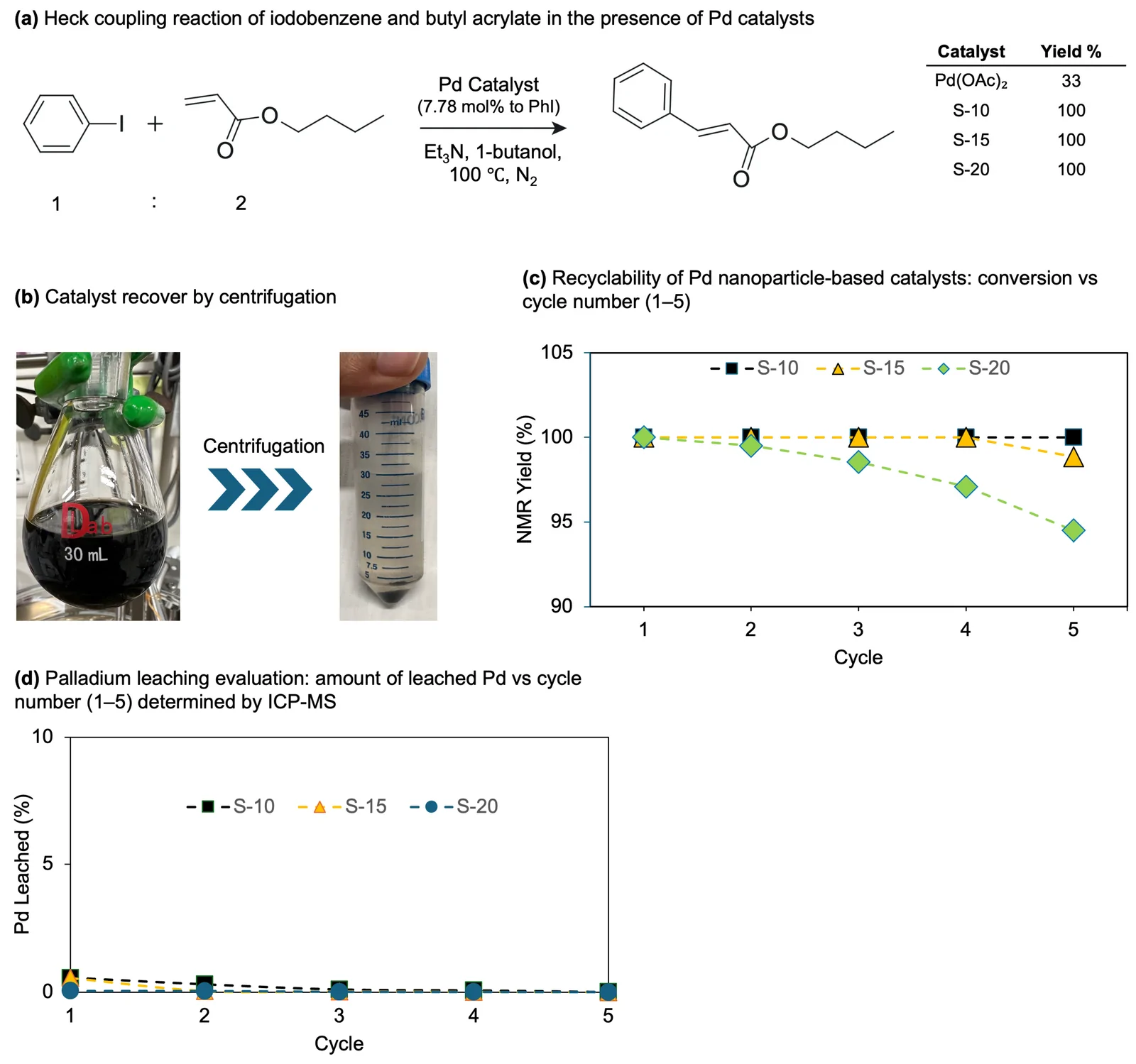
(**a**) Mizoroki–Heck coupling reaction catalyzed by the embedded PdNPs systems, (**b**) photo images of the reaction mixture after the first-cycle reaction using S-10 and the liquid after catalyst recovery by centrifugation, (**c**) recyclability performance, and (**d**) palladium leaching degrees per cycle number (1–5) determined by ICP-MS (reaction conditions: catalyst load = 77.8 μmol of Pd, iodobenzene = 1.0 mmol, *n*-butyl acrylate = 2.0 mmol, Et_3_N = 2.0 mmol, 1-butanol = 4 mL, 100 °C, 4 h, N_2_, NMR yields were determined by ^1^H NMR spectroscopy).

The Mizoroki–Heck coupling reaction of iodobenzene and *n*-butyl acrylate was performed using the polymer-templated PdNP catalysts and Pd(OAc)_2_ (Figure 4a). The reaction was performed in 1-butanol at 100 °C for 4 h using triethylamine as the base. In the first cycle, all the embedded catalysts (S-10, S-15, and S-20) achieved a >99% yield, whereas Pd(OAc)_2_ resulted in a 33% yield. Figure 4b shows the appearance of the reaction mixture after the first-cycle reaction using S-10 (left) and the liquid after catalyst recovery by centrifugation (right). The catalyst was successfully precipitated by centrifugation at 14,000 rpm (mass of recovered catalyst for five cycles is presented in Table S9).

All three catalysts (S-10, S-15, and S-20) maintained >95% NMR yield over five consecutive cycles (Figure 4c and Figures S11–S13). Specifically, S-10 achieved a quantitative yield even at the fifth cycle. The excellent cycle stability was manifested by negligible leaching during the catalytic process (Figure 4d). The ICP-MS analysis of post-reaction supernatants after centrifugation revealed that the supernatants contained sub-microgram Pd in ppm-order concentrations, whereas the Pd amounts used in this catalytic cycle were 9.0–9.2 mg (Table S7 represents the actual palladium contents in fresh catalyst, and Table S8 shows the percentage of Pd leached after each cycle). This excellent recovery of the catalysts led to the quantitative catalytic reaction during the repeated use. The loss of catalytic performance over repeated cycles became significant as the decrease in the relative composition of the polymer to Pd. This behavior suggests that insufficient polymer coating led to surface oxidation of PdNPs.

The DLS profiles of the recycled catalysts exhibited narrower hydrodynamic size distributions than those of the fresh, unreacted catalysts, while preserving both the swollen microgel network and primary sub-10 nm Pd nanoparticles (Figure S9). Notably, repeated cycling at 100 °C did not induce thermal fatigue, leading to polymer collapse, while preventing nanoparticle aggregation into macroclusters. This structural robustness over successive reaction cycles is consistent with the soft, dynamic character of the polymer–metal assembly. Under the thermal reaction conditions (100 °C), catalytically active Pd species can transiently dissociate from the carboxyl-functionalized copolymer template into the local reaction medium to mediate the Mizoroki–Heck coupling (“release”). Upon completion of the reaction and subsequent cooling, these Pd species are re-coordinated and re-encapsulated by the amphiphilic SMA-*g*-polyether matrix (“catch”), restoring the reassembled microgel network with negligible metal leaching into the supernatant (Figure 4d and Tables S8 and S9). This dynamic “release-and-catch” mechanism rationalizes the sustained quantitative yields across consecutive cycles and supports the quasi-homogeneous behavior of this soft heterogeneous catalytic system.

The recyclability results further support the coordination of the carboxylate moieties in SMA-*g*-polyether is sufficiently robust to retain PdNPs within the network under the reaction conditions. This finding is particularly noteworthy because it demonstrates that a system without covalent cross-linkage exhibits stability comparable to that of conventional cross-linked catalysts.

Furthermore, the substrate scope was expanded using electron-deficient and electron-rich aryl iodides. Electron-deficient 1-iodo-4-nitrobenzene resulted in 100% NMR conversion to *n*-butyl 4-nitrocinnamate using S-10, S-15, and S-20, while electron-rich 1-iodo-4-methoxybenzene afforded *n*-butyl 4-methoxycinnamate in 75.2% (S-10), 88.5% (S-15), and 85.1% (S-20) NMR yields (Figures S16 and S17, Tables S5 and S6).

### 3.3. Life Cycle Assessment of Environmental Impact

To evaluate the environmental advantage, a life cycle assessment was conducted by calculating the global warming potential (GWP) and energy consumption. The GWP is expressed in kg CO_2_-eq and measures the greenhouse gas emissions associated with solvent production, palladium mining, and heating. Energy consumption is expressed in MJ and measures the total energy demand for the same processes. As a conventional heterogeneous Pd-catalyzed Mizoroki–Heck reaction, a reaction system conducted in DMF at 120 °C catalyzed by a porous polymer bearing Pd salen catalyst moieties was selected [43]. The following parameters were calculated: GWP_DMF_, GWP_BuOH_, net GWP reduction, energy for the DMF system (E_DMF_), energy for the butanol system (E_BuOH_), and net energy reduction. The conventional DMF-based system and the butanol-based system (present system) were compared using these parameters. The reaction using S-10 in butanol at 100 °C achieved Pd loss less than 1% during the five cycles of the centrifugation-assisted recovery (Table 2 and Figure 4), whereas the previously reported system leaches 4.6 wt% of Pd.

For the calculation, we used published LCA data for DMF [44], biobased butanol [56], palladium mining and refining [57], and Japan’s electricity mix [58]. The functional unit was a single reaction batch (100 mL solvent, 10.2 mg Pd, 100 °C, 4 h). The heating power was assumed to be 100 W, resulting in 0.4 kWh = 1.44 MJ and 0.174 kg CO_2_-eq based on Japan’s grid electricity (0.435 kg CO_2_-eq per kWh).

The elimination of palladium loss represents another environmental advantage, although the contribution of palladium to the total GWP of the DMF system is relatively small because of its use in catalytic amounts (0.00115/0.487 = 0.24%). Palladium is a rare precious metal, and its mining and refining require approximately 72,000 MJ per kilogram and emit 2500 kg CO_2_-eq per kilogram [59]. However, because the actual loss per batch is only 4.6% of the 10 mg loading, the per-batch impact is modest, whereas this system with negligible loss is advantageous over non-recyclable catalytic systems, which correspond to 0.025 kg CO_2_-eq (the mass of catalyst recovered after each cycle is presented in Table S9). emission and 0.720 MJ energy loss by wasting palladium. The more significant benefit of this recoverable system becomes apparent over multiple cycles (Table 3).

The numerical results can be expressed by the following equations, where GWP = global warming potential (kg CO_2_-eq), m = mass (kg), EF = emission factor (kg CO_2_-eq per kg or per kWh), P = heating power (kW), t = reaction time (h), and ED = energy demand (MJ per kg).

GWP for DMF system:

(1) $${\mathrm{GWP}}_{\mathrm{DMF}}=(m_{\mathrm{solvent},\mathrm{DMF}}\times {\mathrm{EF}}_{\mathrm{DMF}})+(m_{\mathrm{Pd}}\times \mathrm{loss}\;\mathrm{fraction}\times {\mathrm{EF}}_{\mathrm{Pd}})+(P\times t\times {\mathrm{EF}}_{\mathrm{elec}})$$

GWPDMF=(0.0944×3.3)+(0.00001×0.046×2500)+(0.4×0.435)GWPDMF=0.312+0.00115+0.174GWPDMF=0.487 kg CO2-eq

GWP for butanol system:

(2) $${\mathrm{GWP}}_{\mathrm{butanol}}=(m_{\mathrm{solvent},\mathrm{butanol}}\times {\mathrm{EF}}_{\mathrm{butanol}})+(m_{\mathrm{Pd}}\times 0\times {\mathrm{EF}}_{\mathrm{Pd}})+(P\times t\times {\mathrm{EF}}_{\mathrm{elec}})$$

GWPbutanol=(0.0810×1.8)+(0×2500)+(0.4×0.435)GWPbutanol=0.146+0+0.174GWPbutanol=0.320 kg CO2-eq

Net GWP reduction:

GWPratio=GWPbutanolGWPDMF=0.3200.487=0.657GWPreduction=1−GWPratio=1−0.657=0.343Net GWP reduction=34.3%

Energy for the DMF system:

(3) $$E_{\mathrm{DMF}}=(m_{\mathrm{solvent},\mathrm{DMF}}\times {\mathrm{ED}}_{\mathrm{DMF}})+(m_{\mathrm{Pd}}\times \mathrm{loss}\;\mathrm{fraction}\times {\mathrm{ED}}_{\mathrm{Pd}})+(P\times t\times 3.6)$$

EDMF=(0.0944×92)+(0.00001×0.046×72,000)+(0.4×3.6)EDMF=8.68+0.0331+1.44EDMF=10.15 MJ

Energy for the butanol system:

(4) $$E_{\mathrm{butanol}}=(m_{\mathrm{solvent},\mathrm{butanol}}\times {\mathrm{ED}}_{\mathrm{butanol}})+(0)+(P\times t\times 3.6)$$

Ebutanol=(0.0810×48)+0+1.44Ebutanol=3.89+0+1.44Ebutanol=5.33 MJ

Net energy reduction:

Eratio=EbutanolEDMF=5.3310.15=0.525Ereduction=1−Eratio=1−0.525=0.475Net energy reduction=47.5%

Several limitations of this LCA should be noted. The calculations assume idealized conditions and do not include solvent recovery, purification energy, waste treatment, or the environmental impact of manufacturing the polymer templates. The palladium mining data represent primary production from South African mines and may vary depending on the source and refining method. The electricity mix used (Japan average) assumes grid electricity; using renewable energy would proportionally reduce the GWP of both systems. Nevertheless, the comparison clearly shows that switching from DMF to butanol combined with palladium recovery provides measurable environmental benefits.

Furthermore, the LCA results presented above provide quantitative evidence of the importance of green chemistry in catalyst design. In conventional research, the main focus is often on catalytic activity and selectivity. The environmental impact reduction achieved by solvent selection and recyclability improvement has often been overlooked. The impact of the 34.3% GWP reduction per batch in the present system will be more pronounced in large-scale production. Palladium is designated as a critical raw material in many countries because of its supply risk and mining intensity. Therefore, the improvement in recovery efficiency has strategic importance beyond simple cost savings.

## 4. Conclusions

A graft copolymer-templated PdNP catalyst for the Mizoroki–Heck reaction, with high activity and reusability, was developed via interparticle connection of PdNPs within a polymer matrix bearing carboxy groups and grafted polyether chains. The carboxy group produced seeds for the growth of PdNPs, facilitated by mild reduction with methanol, and served to connect the formed PdNPs. The densely grafted polyether chains facilitated high dispersibility in greener 1-butanol, thereby maintaining high catalytic activity and controlled growth of PdNPs during synthesis. The environmental effectiveness was supported by LCA.

S-10, a PdNP catalyst with the highest polymer matrix composition, showed excellent recyclability, indicating that a sufficient amount of polymer is essential for maintaining the dispersion and suppressing deactivation and leaching. The combination of an amphiphilic polyether segment and coordination capability provides a unique environment that enables the high dispersion of PdNPs and efficient access of the reactants to the catalytic sites. From this perspective, the polymer-templated PdNP catalyst system can be regarded as a soft and dynamic heterogeneous catalyst exhibiting quasi-homogeneous characteristics via the “release-and-catch” mechanism. This approach offers an effective strategy for designing advanced catalytic materials without relying on covalent crosslinking, highlighting the importance of controlling polymer architecture in the development of functional nanoreactors. Extending this catalyst to other reactions is expected to provide useful insights into the applicability of this design strategy.

## Figures and Tables

**Table 1:** Formulation of SMA-*g*-polyether-supported PdNP catalysts.

Catalyst †	Polymer Stock Solution * (mL)	Pd(OAc)_2_ (mg)	THF (mL)	Pd/Anhydride ‡ (mol)
S-10	40	102.8	20	10:1
S-15	40	154.2	20	15:1
S-20	40	205.6	20	20:1

† Catalyst nomenclature: S-x, where x denotes the molar feed ratio of Pd(OAc)_2_ to maleic anhydride-derived repeat units used in the catalyst preparation. * Each 40 mL aliquot of polymer stock solution (2.5 mg mL^−1^ in methanol) corresponds to 100 mg of SMA-*g*-polyether. ‡ Pd/Anhydride feed ratio: Calculated assuming complete grafting of each maleic anhydride unit with one Jeffamine M-2070 chain (average molecular weight ≈ 2000 g mol^−1^), corresponding to an effective grafted repeat-unit molecular weight of approximately 2200 g mol^−1^. This assumption was used solely for the stoichiometric calculation of the Pd(OAc)_2_ feed.

**Table 2:** Environmental impact comparison between conventional DMF system and present butanol system per reaction batch (100 mL solvent, 10.2 mg Pd, 4 h).

Impact Category	DMF System	Butanol System	Reduction
Solvent GWP (kg CO_2_-eq)	0.312	0.146	53.20%
Solvent energy (MJ)	8.68	3.89	55.20%
Pd loss GWP (kg CO_2_-eq)	0.00115	0	100%
Pd loss energy (MJ)	0.0331	0	100%
Heating GWP (kg CO_2_-eq)	0.174	0.174	0%
Heating energy (MJ)	1.44	1.44	0%
Total GWP (kg CO_2_-eq)	0.487	0.32	34.30%
Total energy (MJ)	10.15	5.33	47.50%

The most substantial individual improvement was observed for solvents. Butanol has a 53.2% lower GWP and 55.2% lower energy demand than DMF. This is primarily because of the lower carbon footprint (1.8 vs. 3.3 kg CO_2_-eq per kg) and lower energy demand (48 vs. 92 MJ per kg) of biobased butanol than those of DMF.

**Table 3:** Cumulative environmental savings over multiple reaction cycles. Calculations assume 4.6% Pd loss per batch for the DMF system and 0% Pd loss for the butanol system.

Number of Cycles	DMF System Cumulative GWP (kg CO_2_-eq)	Butanol System Cumulative GWP (kg CO_2_-eq)	Cumulative Savings (kg CO_2_-eq)
1	0.487	0.32	0.167
5	2.435	1.6	0.835
10	4.87	3.2	1.67

## Data Availability

The original contributions presented in this study are included in the article/Supplementary Materials. Further inquiries can be directed to the corresponding author.

## Supplementary Materials

The following supporting information can be downloaded at: https://www.mdpi.com/article/10.3390/ma19214434/s1, Figure S1: ^1^H NMR spectrum of poly(styrene-co-maleic anhydride) (SMA) (500 MHz, DMSO-d_6_); Figure S2: ^1^H NMR spectrum of Jeffamine M-2070 (600 MHz, DMSO-d_6_); Figure S3: ^1^H NMR spectrum of SMA-g-polyether (600 MHz, CDCl_3_); Figure S4: FTIR spectra of SMA, Jeffamine M-2070, and SMA-g-polyether; Figure S5: TEM images of (a) S-10, (b) S-15, and (c) S-20 (scale bar = 50 nm); Figure S6: BF-STEM images of (a) S-10, (b) S-15, and (c) S-20 (all at 250,000× magnification), and (e) S-20 (500,000× magnification); (d) SEM image of S-10 (100,000× magnification); Figure S7: XRD patterns of S-10, S-15, and S-20 (Cu Kα radiation); Figure S8: DLS hydrodynamic size distributions of fresh catalysts S-10, S-15, and S-20; Figure S9: DLS hydrodynamic size distributions of recovered catalysts S-10, S-15, and S-20 after five catalytic cycles; Figure S10: ^1^H NMR spectra (500 MHz, DMSO-d_6_) of the crude mixtures from the first-cycle Mizoroki–Heck reaction of iodobenzene and n-butyl acrylate using S-10; Figure S11: ^1^H NMR spectra (600 MHz, CDCl_3_) of the crude mixtures from the Mizoroki–Heck reaction of iodobenzene and n-butyl acrylate over cycles 1–5 using catalyst S-10; Figure S12: ^1^H NMR spectra (600 MHz, CDCl_3_) of the crude mixtures from the Mizoroki–Heck reaction of iodobenzene and n-butyl acrylate over cycles 1–5 using catalyst S-15; Figure S13: ^1^H NMR spectra (600 MHz, CDCl_3_) of the crude mixtures from the Mizoroki–Heck reaction of iodobenzene and n-butyl acrylate over cycles 1–5 using catalyst S-20; Figure S14: ^1^H NMR spectrum (500 MHz, CDCl_3_) of isolated n-butyl cinnamate; Figure S15: Quantitative ^1^H NMR spectra (600 MHz, CDCl_3_) of the control reaction mixture (without n-butyl acrylate or Pd catalyst), with the dimethyl sulfone (DMS) signal at 3.633 ppm normalized to 100: (a) after 12 h at 100 °C and (b) at 0 h and 25 °C; Figure S16: ^1^H NMR spectra (600 MHz, CDCl_3_) of the crude mixtures from the Mizoroki–Heck reaction of 1-iodo-4-nitrobenzene and n-butyl acrylate catalyzed by S-10, S-15, and S-20; Figure S17: ^1^H NMR spectra (500 MHz, CDCl_3_) of the crude mixtures from the Mizoroki–Heck reaction of 1-iodo-4-methoxybenzene and n-butyl acrylate catalyzed by S-10, S-15, and S-20; Table S1: Quantitative ^1^H NMR integration analysis of Jeffamine M-2070 (DMSO-d_6_); Table S2: Quantitative ^1^H NMR integration analysis of SMA-g-polyether (Jeffamine M-2070) (CDCl_3_); Table S3: XRD acquisition and data-processing parameters for S-10, S-15, and S-20; Table S4: XRD peak parameters and Scherrer crystallite-size estimates for S-10, S-15, and S-20; Table S5: Residual iodobenzene integral ratios (7.33 ppm, 2H) relative to the vinylene proton signal of n-butyl cinnamate (7.68 ppm, 1H), and the corresponding ^1^H NMR yields of n-butyl cinnamate over five consecutive Heck reaction cycles using S-10, S-15, and S-20 catalysts; Table S6: ^1^H NMR integration ratios and calculated NMR yields for the Mizoroki–Heck reaction of 1-iodo-4-methoxybenzene with n-butyl acrylate using S-10, S-15, and S-20; Table S7: ICP-MS determination of palladium content in digests of fresh polymer-supported catalysts S-10, S-15, and S-20; Table S8: Palladium leaching by catalyst and recycling cycle during Heck coupling reactions, determined by ICP-MS using the standard addition method (^106^Pd, dual-detector mode, n = 3); Table S9: Mass of catalyst recovered after each reaction cycle.
