# Supplementary material for: Amphiphilic Graft Copolymer-Templated Pd Nanoparticles: A Highly Recyclable Catalyst for Greener Mizoroki–Heck Reaction
Source: Materials (Basel). 2026 Oct 22;19(21):4434. doi: 10.3390/ma19214434 (PMC13764454; doi:10.3390/ma19214434)
Supplement: Supplementary file 1 [file materials-19-04434-s001.zip › materials-4386366-supplementary.pdf]

Supplementary Materials

Amphiphilic Graft Copolymer-Templated Pd Nanoparticles: A Highly Recyclable Catalyst for Greener Mizoroki–Heck Reaction

Muhammad Faizan Ali and Bungo Ochiai

Table of Contents

1. Materials and Methods
2. Synthesis and Characterization of SMA-g-Polyether
3. Preparation and Characterization of Pd Nanoparticles
4. Catalytic Performance in Mizoroki–Heck Reaction
5. Substrate Scope
6. Catalyst Recycling and Stability
7. ICP-MS Analysis

1 Materials and Methods

All reagents and solvents were obtained from commercial sources and used without further purification unless otherwise stated.

Instrumentation:

^{1}H NMR: JEOL JNM-ECZ500R (500 MHz) and JEOL JNM-ECZ600R (600 MHz) spectrometers

FTIR: PerkinElmer Spectrum Two spectrometer (ATR mode)

TEM: JEOL JEM-2100F (200 kV, point resolution 0.23 nm)

STEM: JEOL JSM-IT800 (30 kV, working distance 5 mm)

XRD: Rigaku MiniFlex diffractometer (Cu Kα, 30 kV, 15 mA)

DLS: Malvern Zetasizer Nano ZS (4 mW He–Ne laser, λ = 633 nm, 173° backscatter)

ICP-MS: Agilent 7900 (^{106}Pd isotope, dual detector mode)

2 Synthesis and Characterization of SMA-g-Polyether

2.1 ^{1}H NMR of Poly(styrene-co-maleic anhydride) (SMA)

Figure S1 shows the ^{1}H NMR spectrum of poly(styrene-*co*-maleic anhydride) (SMA) recorded in DMSO-d_6, highlighting the phenyl (Ar–H) and backbone aliphatic (methine/methylene) proton regions.

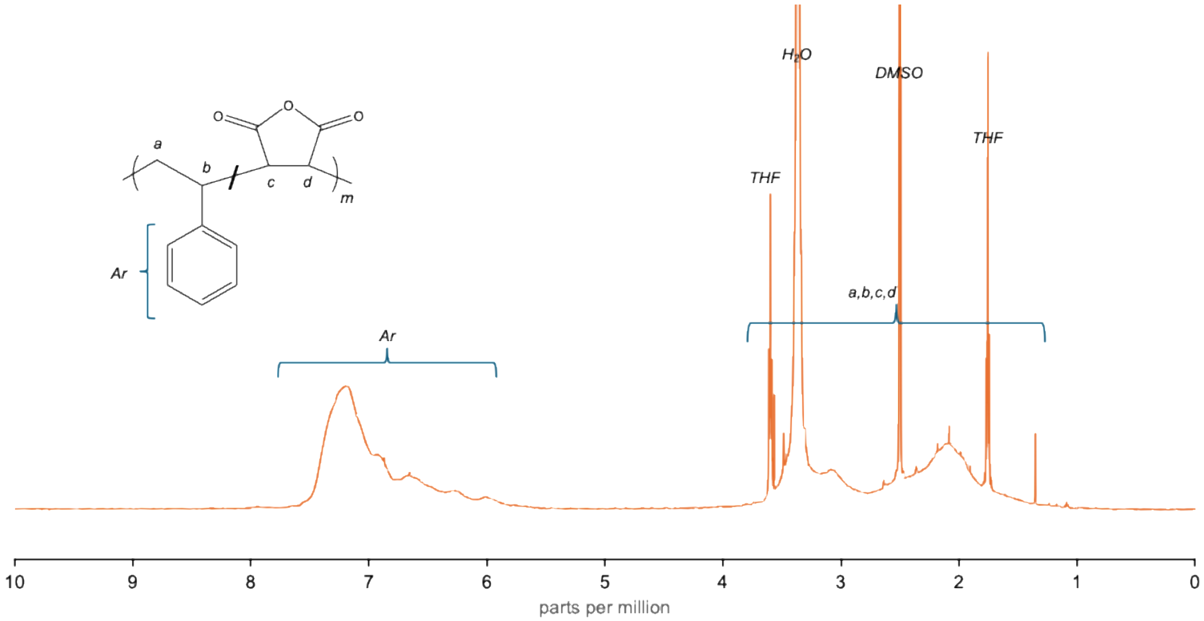

Figure S1: ^{1}H NMR spectrum of poly(styrene-*co*-maleic anhydride) (SMA) (500 MHz, DMSO-d_6).

2.2 ^{1}H NMR of Jeffamine M-2070

Figure S2 presents the ^{1}H NMR spectrum of Jeffamine M-2070 in DMSO-d_6, where terminal methoxy (3.24 ppm), polyether backbone (–OCH_2–, –OCH–, 3.20–3.75 ppm), and side chain (1.02–1.06 ppm) and terminal (0.85–0.90 ppm) methyl (–CH_3) resonances are observed.

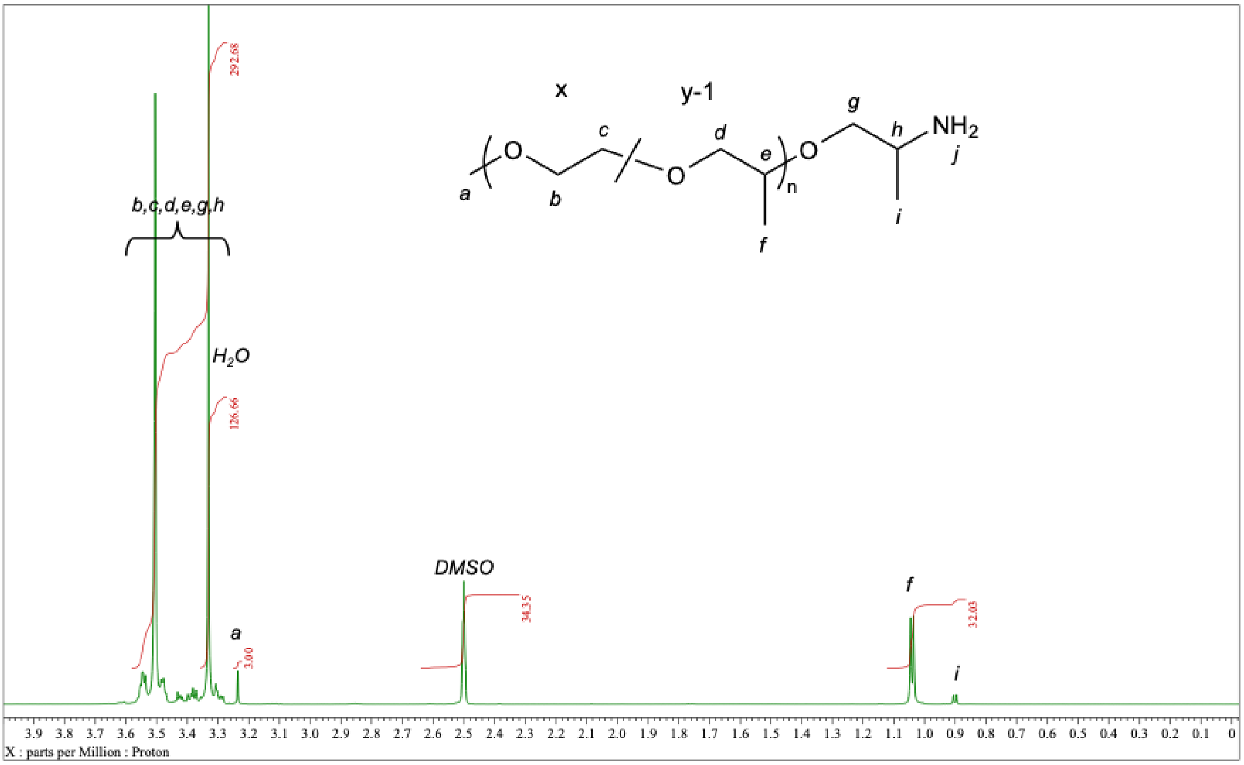

Figure S2*:* ^{1}H NMR spectrum of Jeffamine M-2070 (600 MHz, DMSO-*d*$_6$).

Table S1: Quantitative ^{1}H NMR integral analysis of Jeffamine M-2070 (DMSO-*d*$_6$)

Proton assignment	Chemical shift (ppm)	Normalized integral	Proton type
**Polyether backbone (–OCH_2CH_2–, –$OCH(CH_3)CH_2$–)**	3.20–3.75	166.18*	Methylene + methine
**Poly(propylene glycol) methyl (–CH3)**	~1.10	32.03	Methyl

Characteristic proton ratio

Polyether backbone	PPG methyl	Backbone : methyl ratio
166.18	32.03	**31 : 10**

*Corrected after subtraction of the residual water signal.

2.3 ^{1}H NMR of SMA-g-Polyether

Figure S3 displays the ^{1}H NMR spectrum of SMA-*g*-polyether in $CDCl_3$, confirming successful grafting through the coexistence of the aromatic protons in the styrene unit and the Jeffamine polyether chain. The grafting efficiency (100%) was calculated from the integral ratio of the aromatic protons in the styrene unit (5H) and methyl protons (32.46 H, theoretical value = 32.03H) in the propylene glycol unit in the Jeffamine chain.

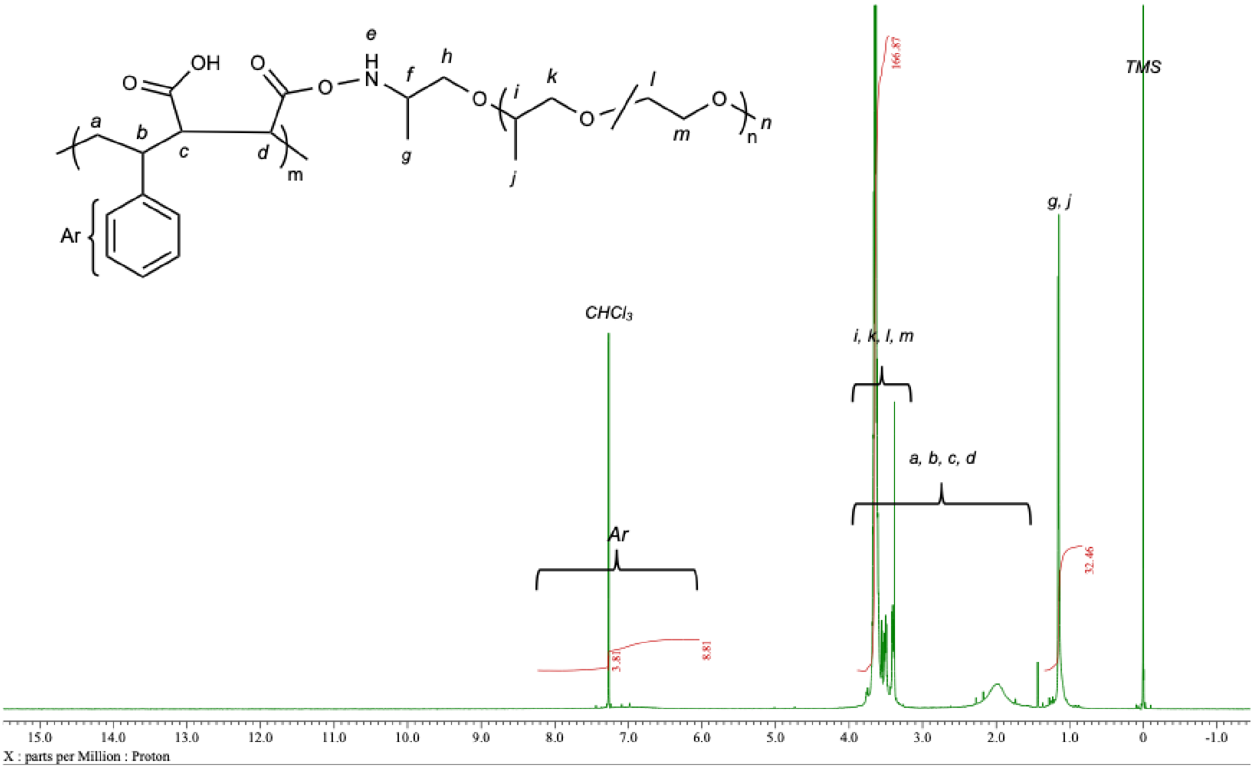

Figure S3: ^{1}H NMR spectrum of SMA-*g*-polyether (600 MHz, $CDCl_3$).

Table S2:Quantitative ^{1}H NMR integral analysis of SMA-*g*-Polyether (Jeffamine M-2070) ($CDCl_3$)

Proton assignment	Chemical shift (ppm)	Normalized integral	Proton type
**Styrene aromatic protons (Ar–H)**	6.0–8.3	5.00	Aromatic (phenyl)
**Polyether backbone (–OCH_2CH_2–, –$OCH(CH_3)CH_2$–)**	3.20–3.75	166.87	Methylene + methine
**Poly(propylene glycol) methyl (–CH_3)**	~1.10	32.46	Methyl

Characteristic proton ratio

Styrene (Ar–H)	Polyether backbone	PPG methyl	Backbone : methyl ratio
5.00	166.87	32.46	**31 : 10**

Equations:

Correction of the DMSO-d₆ backbone integral:

$$I_{\text{PEG}} = 292.84 - 126.66 = 166.18$$

Correction of the styrene aromatic integral:

$$I_{\text{Ar}} = 8.81 - 3.81 = 5.00$$

Comparison of characteristic proton ratios:

$$\frac{166.18}{32.03} \approx \frac{166.87}{32.46} \approx \frac{31}{10}$$

2.4 FTIR Spectroscopic Analysis

The FTIR spectrum of SMA shows two intense anhydride C=O bands at 1843 and 1773 cm^{-1}. In contrast, the spectrum of SMA-*g*-polyether displays an amide I and amide II absorptions at 1631 and 1721 cm^{-1}, respectively, along with the absence of the anhydride bands. The ether (C–O–C, 1095 cm^{-1}) and aliphatic C–H (2864–2869 cm^{-1}) bands characteristic of Jeffamine M-2070 and broad absorption bands of COO–H and N–H (2500–3700 cm^{-1}) are also observed demonstrating successful ring-opening and amide formation.

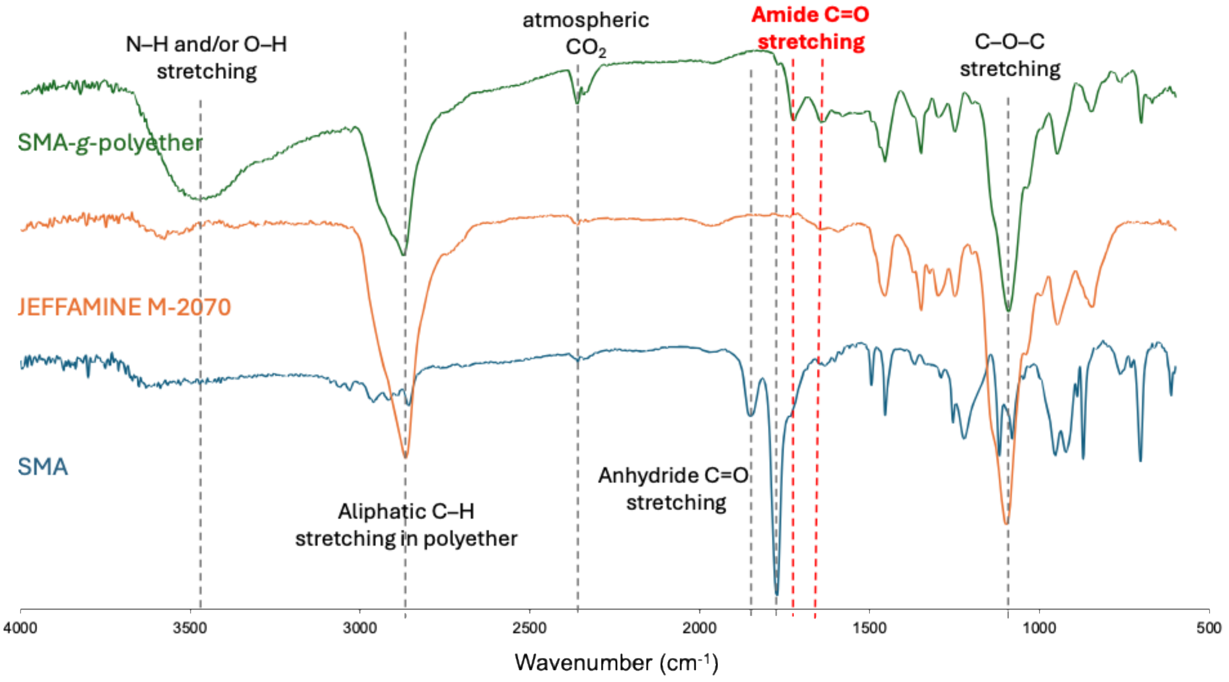

Figure S4: FTIR spectra of SMA, Jeffamine M-2070, and SMA-*g*-polyether.

3 Preparation and Characterization of Pd Nanoparticles

3.1 Transmission Electron Microscopy (TEM)

TEM images of samples S-10, S-15, and S-20 were acquired using a JEM-2100F microscope equipped with a Schottky field-emission electron gun (ZrO/W emitter) operated at an accelerating voltage of 200 kV (Figure S5). The instrument has a point resolution of 0.23 nm and supports magnifications from 50× to 1,500,000×. Imaging was performed using the built-in scanning image observation system with a bottom-mount CCD camera (Orien, Gatan) and/or a side-mount CCD camera (ES500W, Gatan). The polymer matrix is unobservable in these images, and the TEM images reveal the morphology of the Pd-containing domains.

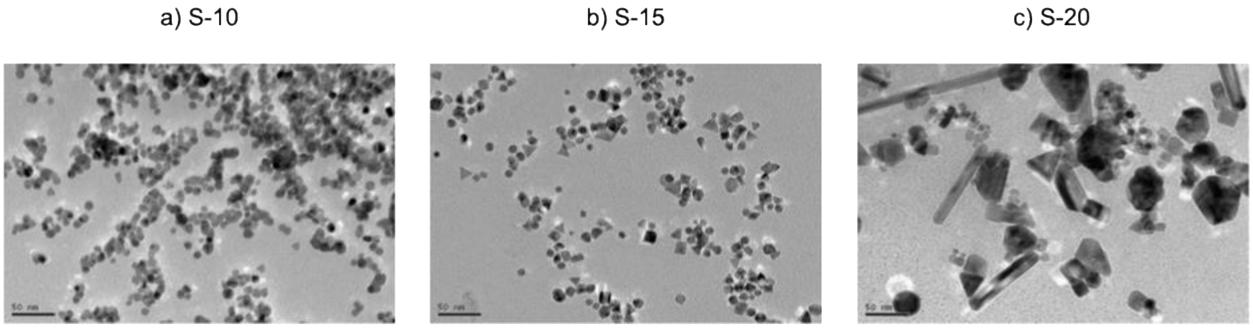

Figure S5: TEM images of (a) S-10, (b) S-15, and (c) S-20 (scale bar = 50 nm).

3.2 STEM imaging of Pd-containing clusters

Bright-field scanning transmission electron microscopy (BF-STEM) images were acquired for samples S-10, S-15, and S-20 (Figures S6) using a JSM-IT800 scanning electron microscope equipped with an in-lens Schottky field-emission electron gun (ZrO/W emitter) at 30 kV accelerating voltage and a working distance of 5 mm. The BF images show brighter polymer-rich regions and darker Pd-rich domains confirm the nanometer-scale features observed by TEM are representative of the broader particle population.

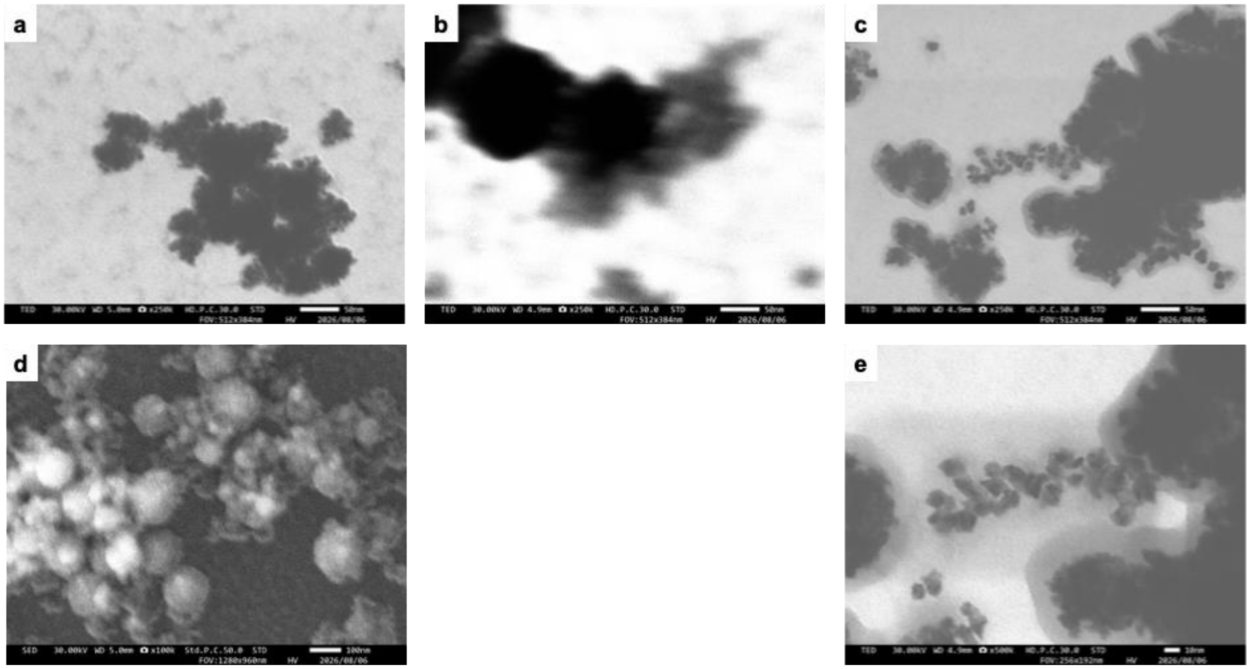

Figure S6: BF-STEM images of (a) S-10, (b) S-15, (c) S-20 (all at 250,000× magnification), and (e) S-20 (500,000× magnification); (d) SEM image of S-10 (100,000× magnification).

3.3 X-ray Diffraction (XRD)

Powder X-ray diffraction (XRD) patterns of S-10, S-15, and S-20 were recorded using a Rigaku MiniFlex diffractometer with Cu Kα radiation at 30 kV and 15 mA. Data were collected over a 2θrange of 2.00–90.00°, using a step size of 0.01°and a scan rate of 2.00 min^{-1}. Measurements were performed using a continuous $\theta/2\theta$scan geometry with a variable divergence slit, a 4.2°scattering slit, and a 0.3 mm receiving slit. The powder samples were poured into the shallow recessed cavity of standard glass sample holders and gently leveled to obtain a flat surface.

The diffraction patterns shown in Figure S9 were generated from the original measured datasets after applying the instrument software's smoothing function. Specifically, a 75-point weighted-average smoothing procedure was applied for graphical presentation. No $K\alpha_2$ removal, background subtraction, baseline correction, peak-search, or peak-fitting procedure was applied. The smoothed patterns are therefore presented only to improve visual readability and were not used to introduce, remove, or relocate diffraction peaks. The machine-readable datasets for smoothed patterns are provided in the Supporting Information as **"XRD Original Data."**

The peaks at 2θ = 40.1°, 46.7°, 68.1°, 81.2°, and 87.3° correspond to the (111), (200), (220), (311), and (222) planes of face-centered cubic Pd(0), respectively.

Table S3: XRD acquisition and data-processing parameters for S-10, S-15, and S-20

Category	Parameter	Experimental condition / procedure
**XRD data acquisition**	Diffractometer	Rigaku MiniFlex diffractometer
	X-ray source	Cu Kα radiation
	Tube voltage/current	30 kV / 15 mA
	X-ray power	450 W
	Scan geometry	Continuous $\theta/2\theta$ scan
	2θ range	2.00 - 90°
	Step size	0.01°
	Scan rate	2.00° min^{-1}
	Divergence slit	Variable
	Scattering slit	4.2°
	Receiving slit	0.3 mm
**Sample preparation**	Sample holder	Standard glass XRD sample holder
	Sample loading	Powder poured into the shallow recessed cavity and gently leveled
**Data processing**	Smoothing	75-point weighted-average smoothing
	Background subtraction	Not applied
	Baseline correction	Not applied
	Intensity normalization	Not applied
	Kα_2 treatment	Not applied
	Intensity reporting	Counts per second (cps)
**Data availability**	Raw-data file	*XRD Original Data*

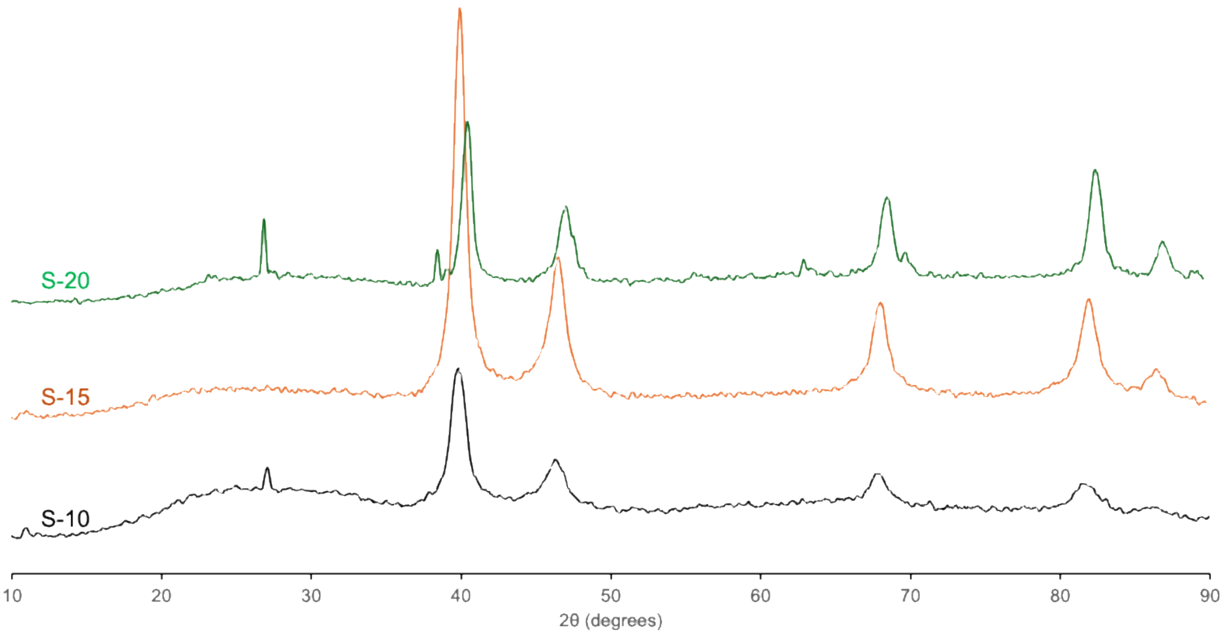

Figure S7: XRD patterns of S-10, S-15, and S-20 (Cu Kα).

Table S4 :XRD peak parameters and Scherrer crystallite-size estimates for samples S-10, S-15, and S-20.

	S-10	S-15	S-20
I_{max}	673.436	1449.820	379.907
**Half intensity, $I_{max}/2$**	336.718	724.910	189.954
**Peak position, 2θ(°)**	39.82	39.93	40.44
**Left half-height position (°)**	38.89	39.34	39.83
**Right half-height position (°)**	40.84	40.46	40.87
**FWHM (°)**	1.95	1.12	1.04
**FWHM (radians)**	0.03403	0.01955	0.01815
**θ (°)**	19.91	19.965	20.22
**Scherrer crystallite size (nm)**	**4.33**	**7.55**	**8.14**

* FWHM was determined from the manually measured half-height positions and converted from degrees to radians. The crystallite size was calculated using the Scherrer equation, $D = K\lambda/(\beta cos\ \theta)$, where $K = 0.90$, $\lambda = 0.15406\ nm$ for Cu Kα_1 radiation, $\beta$is the FWHM in radians, and $\theta = (2\theta)/2$. The reported values are uncorrected for instrumental broadening and should be regarded as approximate coherent crystallite sizes, not necessarily physical nanoparticle diameters.

3.4 Dynamic Light Scattering (DLS) Analysis

Experimental Method: Hydrodynamic size distributions of fresh catalysts (Figure S8) and recovered catalysts after five catalytic cycles (Figure S9) were recorded in methanol at 25 °C using a Zetasizer Nano ZS instrument (Malvern Instruments) equipped with a 4 mW He–Ne laser (λ = 633 nm) and non-invasive backscatter detection at a scattering angle of 173°. Both intensity-weighted and volume-weighted distributions were derived from the raw scattering intensity datasets using the integrated Zetasizer software.

3.4.1 Fresh Catalyst DLS Analysis

Dynamic light scattering inherently yields intensity-weighted hydrodynamic size distributions biased toward larger scattering species owing to the Rayleigh scattering relation ($I \propto d^6$). Consequently, the broad intensity-weighted profiles observed for fresh S-10, S-15, and S-20 reflect the swollen polymer microgel matrix and physical network aggregates.

To resolve smaller populations masked in intensity-weighted plots, volume-weighted profiles were calculated applying the optical parameters of polystyrene latex as an approximation. The volume-weighted profile of fresh S-10 displays a predominant population in the sub-10 nm regime, corresponding to primary, polymer-coated Pd nanoparticles that afford high surface accessibility and catalytic activity. In contrast, S-15 and S-20 exhibit volume-weighted peaks shifted toward larger aggregate sizes. Because these hybrid catalysts comprise organic polymer matrices and metallic Pd nanoparticles with complex optical refractive indices, volume-weighted profiles serve as qualitative ensemble evaluations rather than absolute size measurements.

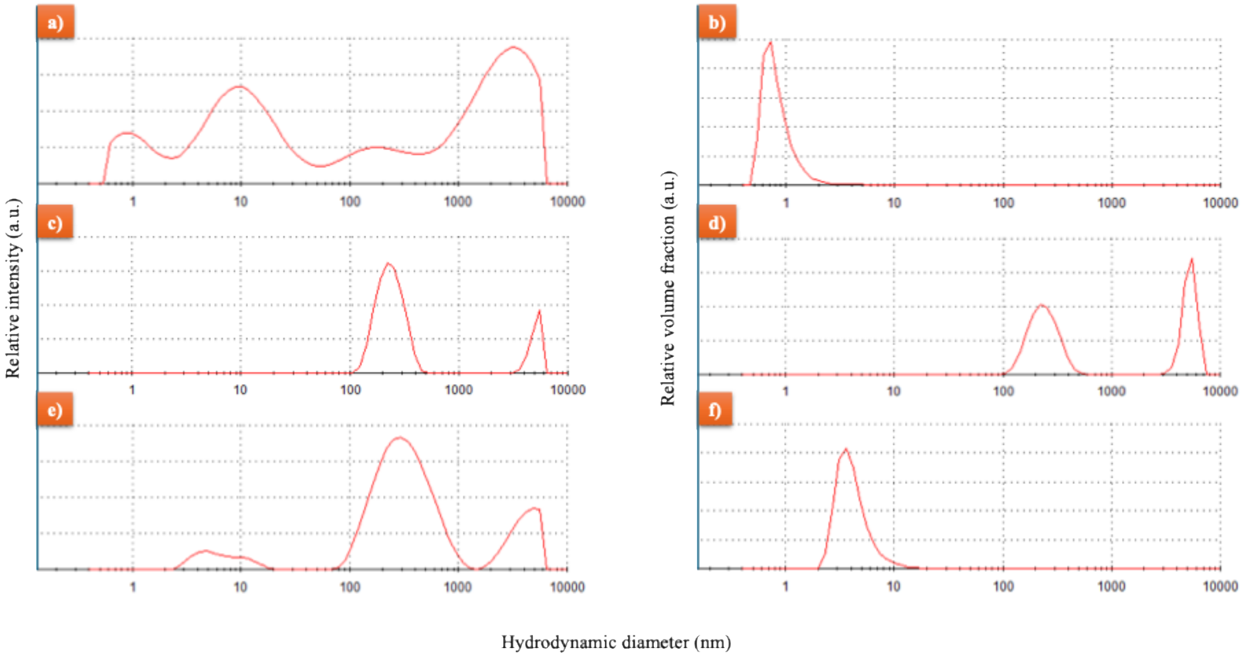

Figure S8: DLS hydrodynamic size distributions of fresh catalysts S-10, S-15, and S-20. Panels (a, c, e) show intensity-weighted distributions, and panels (b, d, f) show the corresponding volume-weighted distributions of S-10, S-15, and S-20, respectively.

3.4.2 Post-Reaction DLS Analysis (Recovered Catalyst)

To evaluate the structural integrity and particle stability of the catalyst during recycling, post-reaction hydrodynamic size analysis of the recovered catalysts (S-10, S-15, and S-20) was conducted after five consecutive reaction cycles (Figure S9).

Post-reaction DLS analysis confirms the retention of both the macroscopic microgel framework and the nanoscale particle dispersion:

Preservation of Microgel Network (Intensity-Weighted Distributions): In panels (a, c, e), the intensity-weighted profiles retain broad micrometer-scale signals. Because light scattering intensity scales with the sixth power of the particle diameter ($I \propto d^6$), these profiles remain dominated by the swollen, physically crosslinked polymer microgel matrix. This confirms that five reaction cycles, centrifugations, and redispersions do not collapse or destroy the swollen polymer network.

Stability of Primary Nanoparticles (Volume-Weighted Distributions): In panels (b, d, f), the volume-weighted distributions—which reduce the disproportionate scattering bias of large species—consistently yield predominant populations below 10 nm. This confirms that the primary polymer-coated Pd nanoparticles within the microgel do not undergo significant particle growth, Ostwald ripening, or macroscopic aggregation into dense metallic precipitates during catalysis.

Together, the dual-weighted DLS profiles demonstrate that the amphiphilic graft copolymer matrix maintains its functional architecture after five cycles: sub-10 nm primary nanoparticles remain protected for high catalytic accessibility, while the swollen microgel assembly is preserved for rapid centrifugal recovery.

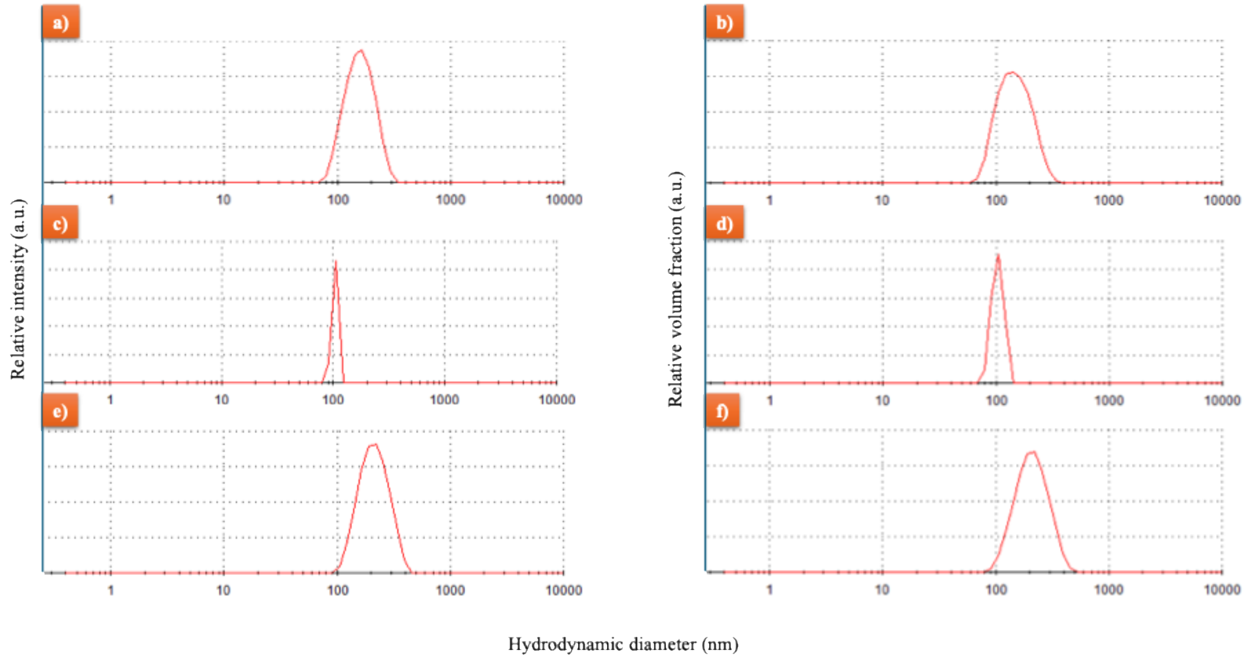

Figure S9: DLS hydrodynamic size distributions of recovered catalysts S-10, S-15, and S-20 after 5 catalytic cycles. Panels (a, c, e) show intensity-weighted distributions, and panels (b, d, f) show the corresponding volume-weighted distributions of S-10, S-15, and S-20, respectively.

4 Catalytic Performance in Mizoroki–Heck Reaction

4.1 General Procedure

In a typical run, iodobenzene (1.0 mmol), *n*-butyl acrylate (1.5 mmol), triethylamine (2.0 mmol), and catalyst (10 mg) in 1-butanol (4 mL) were heated at 100 °C. Reaction progress was monitored by ^{1}H NMR.

4.2 ^{1}H NMR Monitoring of Reaction Progress

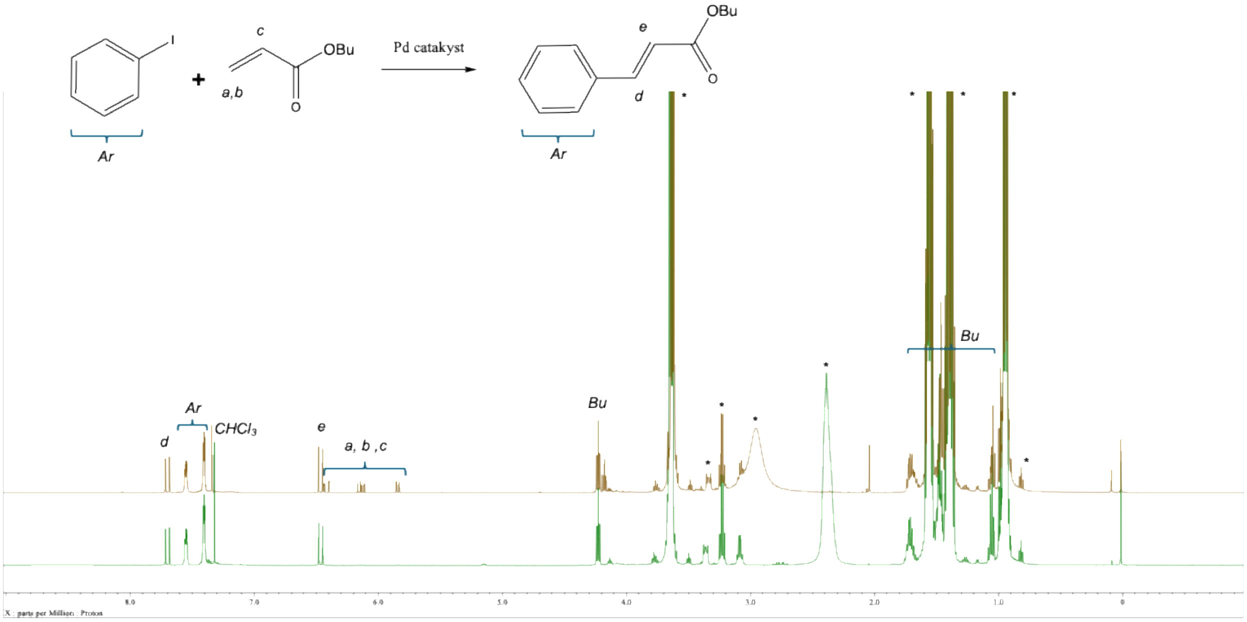

Figure S10: ^{1}H NMR spectra (500 MHz, DMSO-d_6) of the crude mixtures of the first cycle Mizoroki–Heck reaction of iodobenzene and *n*-butyl acrylate using S-10 after 1 (upper) and 4 (lower) hours. The NMR yield was calculated from the integral ratio of the signals of the aromatic protons and the vinylene proton (d) of butyl cinnamate. Asterisks denote the signals of 1-butanol, triethylamine and triethylammonium iodide.

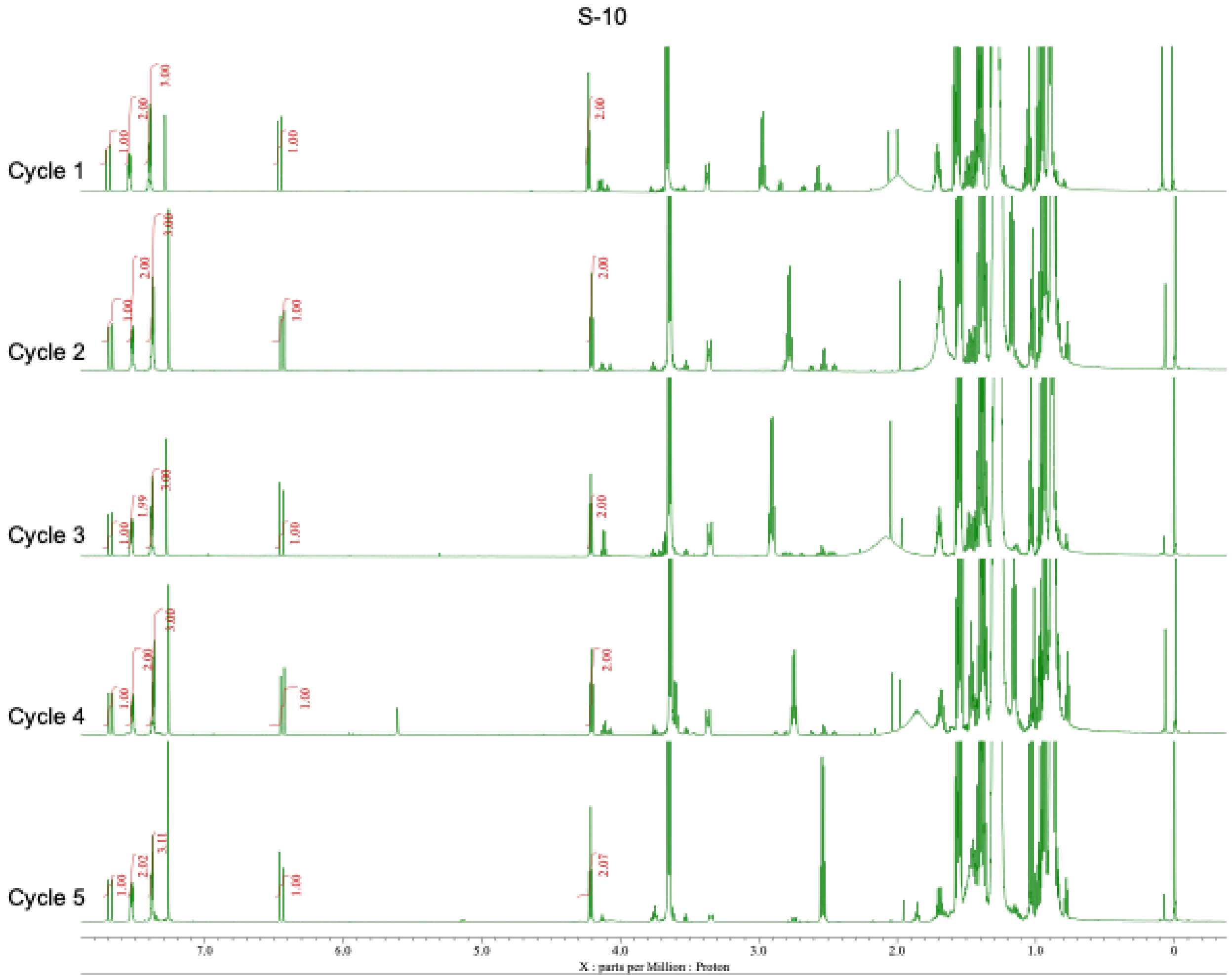

Figure S11: ^{1}H NMR spectra (600 MHz, $CDCl_3$) of the crude mixtures of the Mizoroki–Heck reaction of butyl acrylate and iodobenzene for cycles 1 to 5 using catalyst S-10.

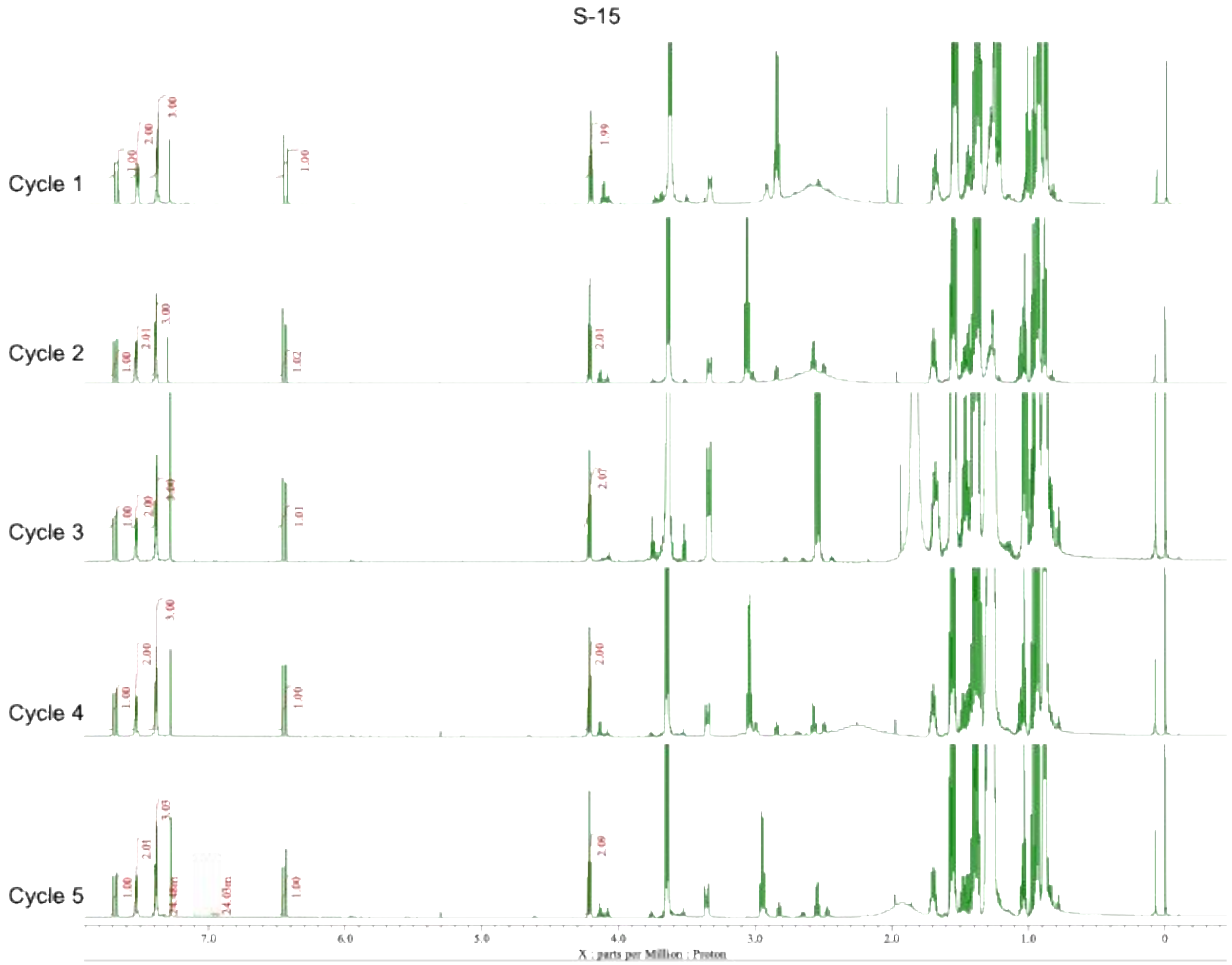

Figure S12: ^{1}H NMR spectra (600 MHz, $CDCl_3$) of the crude mixtures of the Mizoroki–Heck reaction of butyl acrylate and iodobenzene for cycles 1 to 5 using catalyst S-15.

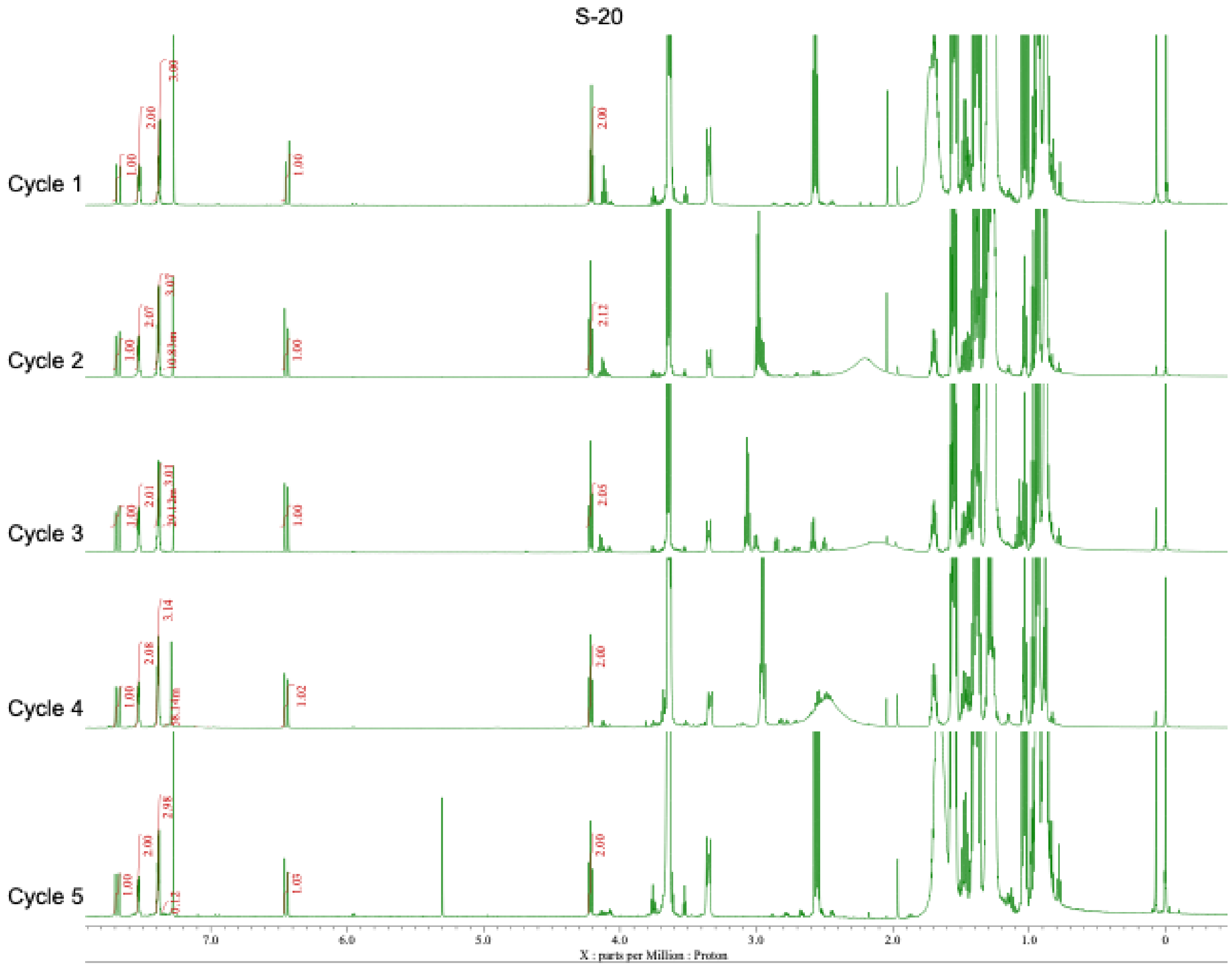

Figure S13: ^{1}H NMR spectra (600 MHz, $CDCl_3$) of the crude mixtures of the Mizoroki–Heck reaction of butyl acrylate and iodobenzene for cycles 1 to 5 using catalyst S-20.

Table S5: Residual iodobenzene integral ratios (7.33 ppm, 2H) relative to vinylene proton of butyl cinnamate (7.68 ppm, 1H), and corresponding ^{1}H NMR yields of butyl cinnamate over five consecutive Heck reaction cycles using S-10, S-15, and S-20 catalysts.

Catalyst	Cycle	Residual iodobenzene integral ratio	^{1}H NMR yield
**S-10**	1	0.01 >	> 99%
**S-10**	2	0.01 >	> 99%
**S-10**	3	0.01 >	> 99%
**S-10**	4	0.01 >	> 99%
**S-10**	5	0.01 >	> 99%
**S-15**	1	0.01 >	> 99%
**S-15**	2	0.01 >	> 99%
**S-15**	3	0.01 >	> 99%
**S-15**	4	0.01 >	> 99%
**S-15**	5	0.025	98.8%
**S-20**	1	0.01 >	> 99%
**S-20**	2	0.010	99.5%
**S-20**	3	0.029	98.5%
**S-20**	4	0.058	97.1%
**S-20**	5	0.11	94.5%
**S-10**	R-1[a]	0.01 >	> 99%
**S-15**	R-1[a]	0.01 >	> 99%
**S-20**	R-1[a]	0.008	99.6%

[a] R-1 denotes the replicated experiments for the 1st cycle

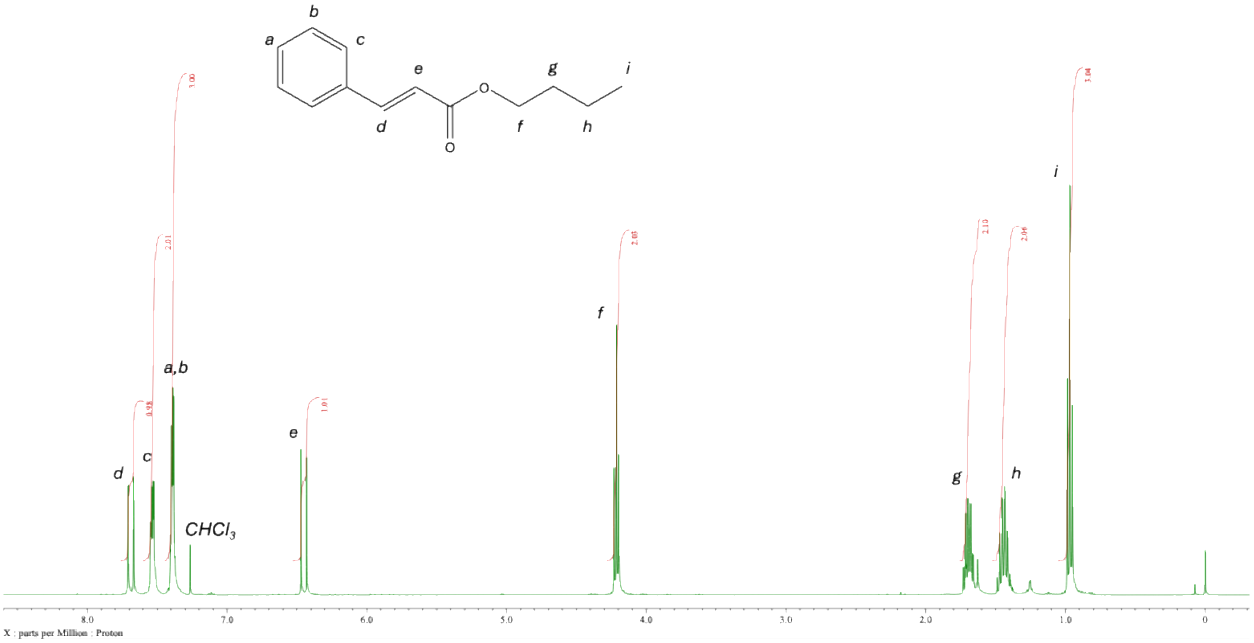

Figure S14: ^{1}H NMR spectrum (500 MHz, $CDCl_3$) of isolated *n*-butyl cinnamate.

4.3 Q-^{1}HNMR spectroscopy: Iodobenzene Stability

To evaluate the stability of iodobenzene under Heck reaction conditions, a dummy reaction was performed in the absence of butyl acrylate and Pd catalyst. The mixture (iodobenzene, 204 mg; triethylamine, 202 mg; 1-butanol, 4

mL; dimethyl sulfone, 9.41 mg) was analyzed by ¹H q-NMR immediately after preparation at 25 °C (0 h) and after heating at 100 °C for 12 h. No significant change in the integral ratios of the aromatic protons of iodobenzene to the methyl protons of dimethyl sulfone was observed, confirming negligible evaporation of iodobenzene at 100 °C and supporting its use as an internal standard for NMR yield calculations.

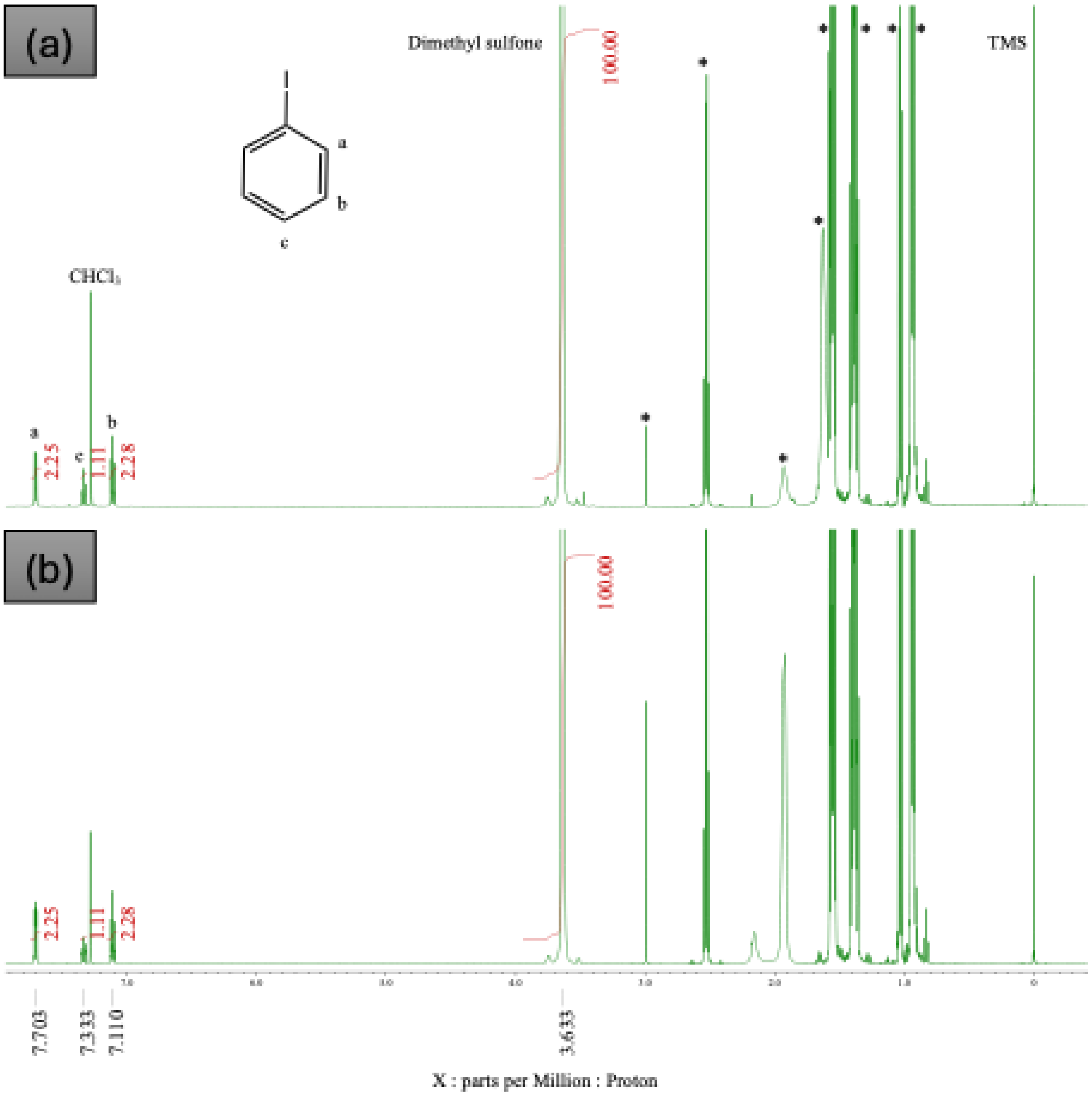

Figure S15: q-¹H NMR spectra (600 MHz, CDCl₃) of the dummy reaction mixture (no butyl acrylate or Pd catalyst) with the dimethyl sulfone (DMS) signal at 3.633 ppm normalized to 100. (a) After 12 h at 100 °C; (b) at 0 h at 25 °C. Asterisks denote signals from 1-butanol, and triethylamine.

5 Substrate Scope

To broaden the substrate scope and evaluate the electronic effects of the aryl iodide, the Mizoroki–Heck reaction was investigated using aryl iodides bearing both electron-withdrawing and electron-donating substituents. In particular, 1-iodo-4-nitrobenzene and 1-iodo-4-methoxybenzene were selected as representative substrates and reacted with *n*-butyl

acrylate under the catalytic conditions using S-10, S-15, and S-20. The use of electronically distinct aryl iodides is a common approach for assessing the functional-group tolerance and generality of Mizoroki–Heck catalysts.

5.1 Electron-withdrawing substrate: 1-Iodo-4-nitrobenzene

The reaction of 1-iodo-4-nitrobenzene with *n*-butyl acrylate proceeded efficiently under the applied conditions. For the reactions catalyzed by S-10, S-15, and S-20, the corresponding *n*-butyl 4-nitrocinnamate was identified by ^{1}H NMR spectroscopy. A quantitative NMR yield of 100% was obtained, demonstrating the excellent activity of the catalysts toward an aryl iodide containing a strongly electron-withdrawing nitro group.

Figure S15 shows the ^{1}H NMR spectra of the crude reaction mixtures. The NMR yield was determined from the integral ratio of the aromatic and vinylene proton signals of the Heck product. Signals arising from 1-butanol, triethylamine, and triethylammonium iodide are marked with asterisks.

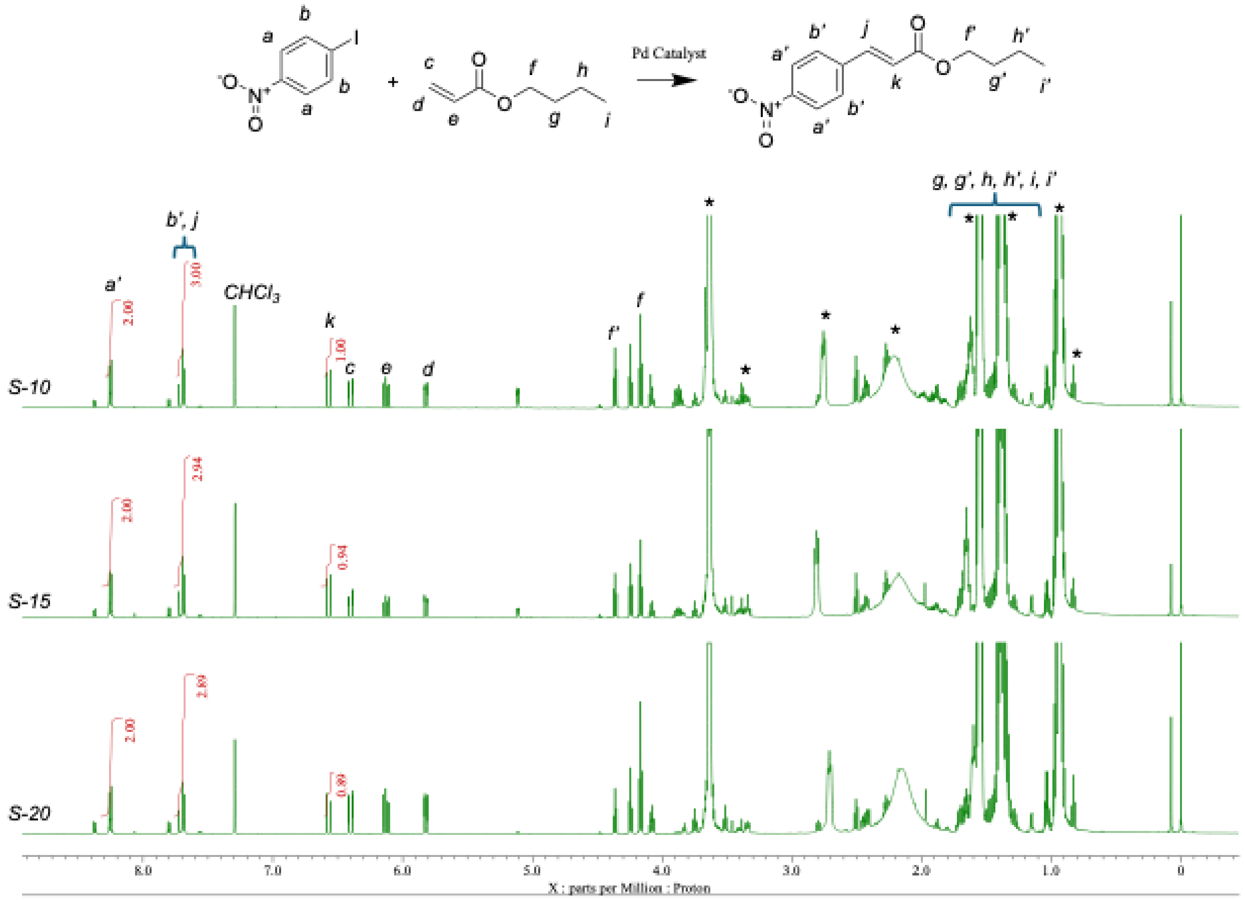

Figure S16: ^{1}H NMR spectra (600 MHz, $CDCl_3$) of the crude mixtures of the Mizoroki–Heck reaction of 1-iodo-4-nitrobenzene and *n*-butyl acrylate catalyzed by S-10, S-15, and S-20. The NMR yield was calculated from the integral ratio of the signals of the aromatic protons and the vinylene protons of butyl cinnamate. Asterisks denote the signals of 1-butanol, triethylamine and triethylammonium iodide.

5.2 Electron-donating substrate: 1-Iodo-4-methoxybenzene

To further examine the substrate scope, 1-iodo-4-methoxybenzene was subsequently employed as an electron-rich aryl iodide. The reaction with *n*-butyl acrylate afforded *n*-butyl 4-methoxycinnamate in moderate to high NMR yields,

depending on the catalyst used. Among the tested catalysts, S-15 provided the highest NMR yield of 88.5%, followed by S-20 at 85.1% and S-10 at 75.2%. These results indicate that the catalytic system tolerates both electron-withdrawing and electron-donating substituents, although the electron-rich 4-methoxy-substituted substrate showed a greater dependence on catalyst composition than the corresponding nitro-substituted substrate.

Figure S16 presents the ^{1}H NMR spectra of the crude reaction mixtures. The product signals were assigned based on the characteristic vinylene protons, aromatic protons, and methoxy proton resonance of *n*-butyl 4-methoxycinnamate.

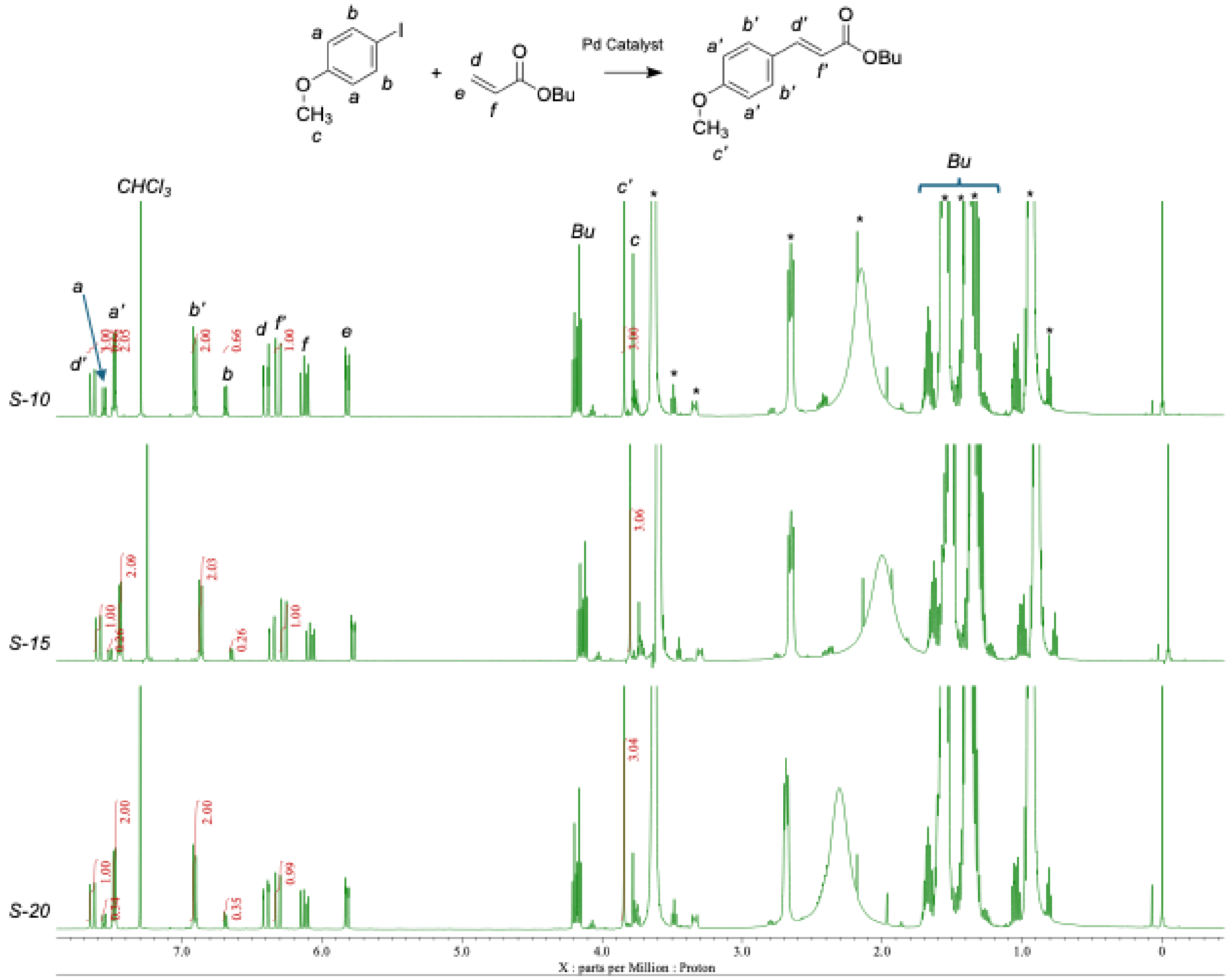

Figure S17: ^{1}H NMR spectra (500 MHz, $CDCl_3$) of the crude mixtures obtained from the Mizoroki–Heck reaction of 1-iodo-4-methoxybenzene with *n*-butyl acrylate catalyzed by S-10, S-15, and S-20. The NMR yield was calculated from the integral ratio of the vinylene proton signals of *n*-butyl 4-methoxycinnamate and the residual 4-iodoanisole signals. Asterisks denote signals arising from 1-butanol, triethylamine, and triethylammonium iodide.

Table S6: ^{1}H NMR integration ratios and calculated NMR yields for the Mizoroki–Heck reaction of 1-iodo-4-methoxybenzene with *n*-butyl acrylate using S-10, S-15, and S-20.

Catalyst	Product d′ / f′ (1H each)	Product a′ / b′ (2H each)	Product c′ (3H)	Residual 4-Iodoanisole d (2H)	Calculated NMR yield
**S-10**	1.00	~2.00	~3.00	0.66	75.2%

**S-15**	1.00	~2.00	~3.00	0.26	88.5%
**S-20**	1.00	~2.00	~3.00	0.35	85.1%

The vinylene proton signals of the product, d′ and f′, were normalized to an integration value of 1.00. The aromatic proton signals, a′ and b′, and the methoxy proton signal, c′, exhibited integration values of approximately 2.00 and 3.00, respectively, confirming the proposed product structure. The NMR yields were calculated from the relative integration of the product vinylene proton signal and the residual aromatic proton signal of 4-iodoanisole.

Overall, these experiments demonstrate that the S-series catalysts are effective for the Mizoroki–Heck coupling of both electron-deficient and electron-rich aryl iodides. The quantitative formation of the nitro-substituted product and the 75.2–88.5% yields obtained with 1-iodo-4-methoxybenzene support the broad substrate tolerance of the catalytic system.

6 Inductively Coupled Plasma Mass Spectrometry (ICP-MS) Analysis

6.1 Fresh Catalyst Analysis

Inductively coupled plasma mass spectrometry (ICP-MS) was performed to determine the Pd content of fresh polymer-supported catalysts (S-10, S-15, and S-20) and to quantify Pd leaching into the reaction supernatants during catalyst recycling over five Heck-reaction cycles.

For fresh-catalyst analysis, a carefully weighed catalyst sample was transferred to a conical flask. Concentrated nitric acid (10 mL) was added, followed by ultrapure water (10 mL). The mixture was heated at 150 °C on a hot plate in a fume hood with stirring until the volume was reduced to less than 10 mL. After cooling to room temperature, the digest was quantitatively transferred to a volumetric flask and diluted with ultrapure water to a final volume of 25.0 mL. The resulting solution was further diluted as required before ICP-MS analysis. The quantitative results are summarized in Table S7.

Table S7: ICP-MS determination of palladium content in fresh polymer-supported catalyst digests (S-10, S-15, S-20).

Catalyst	Sample mass (mg)	Final stock volume (mL)	Dilution ratio	Observed Pd conc. (µg/L)	Actual Pd conc. (µg/L)[a]	Pd mass in digest (mg)[b]	Pd loading (wt%)[c]	Pd per 10 mg catalyst (mg)
S-10	10.85	25.0	10,000	35.916	359,160	8.979	82.76	8.276
S-15	9.81	25.0	10,000	36.88	368,800	9.220	93.99	9.399
S-20	75.52	25.0	500,000	5.9808	2,990,400	74.76	99.00	9.900

[a] Actual Pd conc. (µg/L) = Observed Pd conc. (µg/L) x Dilution ratio

[b] Pd mass in digest (mg) = (Actual concentration in µg/L) × (Final stock volume in mL) × (1 L / 1000 mL) × (1 mg / 1000 µg)

[c] Pd loading (wt%) = Pd mass in digest (mg) / Sample mass (mg)

6.2 Pd Leaching Analysis

Residual Pd concentrations in the post-reaction supernatants were also measured to evaluate catalyst stability, possible homogeneous contributions, and potential product contamination. Before digestion, the organic phase was removed using a rotary evaporator. Concentrated nitric acid (10 mL) and ultrapure water (10 mL) were then added to each residue. The mixture was heated at 150 °C on a hot plate with vigorous stirring until the volume was reduced to less than 10 mL. After cooling to room temperature, the digest was diluted with ultrapure water to a final volume of 25.0 mL. A catalyst-free control was prepared using all Heck-reaction reagents except the Pd catalyst. The quantitative results for Pd leaching are summarized in Table S8. The catalyst-free control showed a background Pd concentration of 1.0 µg/L (0.001 mg/L), indicating minimal contamination.

Table S8: Pd leaching by catalyst and recycling cycle in Heck coupling reactions (ICP-MS, standard addition method, ^{106}Pd, dual detector mode, n = 3).

Catalyst	Cycle	Dilution	Observed Pd (µg/L)	Actual Pd (µg/L)	Actual Pd (mg/L)	Pd leached (mg)	Catalyst used (mg)[a]	Initial Pd amount (mg)[b]	Pd Leached (% of initial Pd)[c]
**S-20**	1	10	18.147	181.5	0.182	0.00455	10.4	8.6070	0.0528
**S-20**	2	10	17.239	172.4	0.172	0.00430	9.9	8.1932	0.052
**S-20**	3	10	3.838	38.4	0.038	0.00095	9.8	8.1105	0.012
**S-20**	4	10	2.404	24.0	0.024	0.00060	9.8	8.1105	0.0074
**S-20**	5	2	0.956	1.9	0.002	0.00005	9.7	8.0277	0.00059
**S-15**	1	1,000	2.179	2179.0	2.179	0.05448	10.5	9.8690	0.552
**S-15**	2	10	16.971	170.0	0.170	0.00425	9.8	9.2110	0.046
**S-15**	3	10	10.916	109.0	0.109	0.00273	9.6	9.0230	0.030
**S-15**	4	10	5.092	50.9	0.051	0.00128	9.6	9.0230	0.014
**S-15**	5	2	1.233	2.5	0.003	0.00006	9.4	8.8351	0.00071
**S-10**	1	500	4.527	2264.0	2.264	0.05660	10.2	10.0980	0.561
**S-10**	2	500	2.381	1191.0	1.191	0.02978	9.8	9.7020	0.31
**S-10**	3	10	41.920	419.0	0.419	0.01048	9.7	9.6030	0.11
**S-10**	4	10	28.658	287.0	0.287	0.00718	9.7	9.6030	0.075
**S-10**	5	10	4.597	46.0	0.046	0.00115	9.6	9.5040	0.012
**Control**	-	2	0.497	1.0	0.001				

[a] Catalyst used (mg) = amount of catalyst used in Mizoroki–Heck reaction
[b] Pd used (mg) = Catalyst used (mg) x Pd loading (wt %)
[c] Pd Leached (%) = Actual Pd leached(mg) / Pd used (mg) x100

Note: Actual Pd (µg/L) = Observed Pd (µg/L) × Dilution. Actual Pd (mg/L) = Actual Pd (µg/L) ÷ 1,000. ICP-MS conditions: ^{106}Pd isotope, dual detector mode, dwell time 35 ns, 3 replicates per sample, standard addition calibration (0, +5, +10 µg/L Pd spikes).

7 Catalyst Recycling and Stability

7.1 Catalyst Recovery

Table S9: Mass of catalyst recovered after each cycle

Catalyst	Cycle 1 (mg)	Cycle 2 (mg)	Cycle 3 (mg)	Cycle 4 (mg)	Cycle 5 (mg)
**S-10**	10.2	9.8	9.7	9.7	9.6
**S-15**	10.5	9.8	9.6	9.6	9.4
**S-20**	10.4	9.9	9.8	9.8	9.7

Experimental error and precision. Catalyst masses were recorded using an analytical balance with a precision of ±0.1 mg. The slight mass fluctuations observed for S-10 (recovery >96% over five cycles) are attributed to minor physical losses during decanting of the supernatant and transfer between vessels. To minimize the contribution of residual triethylammonium iodide salts or adsorbed organic products to the recovered mass, the catalyst was subjected to a rigorous triple-washing cycle with a methanol/diethyl ether (3:1, v/v) mixture (15 mL × 3) followed by drying under vacuum for 6 h.

Corresponding Author

E-mail: ochiai@yz.yamagata-u.ac.jp

ORCID

Muhammad Faizan Ali: https://orcid.org/0000-0002-3611-9025

Notes

The authors declare no competing financial interest.
